# Smart neural network and cognitive computing process for multi task nuclei detection segmentation and classification in breast cancer histopathology images

**DOI:** 10.1038/s41598-025-02575-x

**Published:** 2025-05-26

**Authors:** M. Suriya Begum, S. Kalaivani

**Affiliations:** School of Computer Science Engineering and Information Systems, VIT, Vellore, India

**Keywords:** Histopathology images, Nuclei segmentation, Deep learning, Transfer learning, Breast cancer, Classification model, Cancer, Breast cancer

## Abstract

The detection, segmentation, and differentiation of benign and malignant nuclei from the histopathology images is a challenging task for the early diagnosis of breast cancer. Misinterpretation of True Negative (TN) and False Positive (FP) can generate incorrect results. The proposed Cognitive Computing Process (CCP) detects and segments the nuclei using Deep U-Net with Spatial Attention Mechanisms (SAM) and microns-per-pixel measurements to accurately locate and assess nuclei density. To separate the nuclei of benign and malignant, the patches are introduced to leverage the model’s learning process. The proposed Smart Neural Network (SNN) models contain Smart Convolutional Neural Network (SCNN) and Deep Convolutional Neural Network (DCNN) to reduce incorrect results. Proposed CCP and SNN were evaluated using the BreakHis dataset, which contains 5547 images of benign and malignant samples at various magnifications (40×, 100×, 200×, 400×). These images were processed into patches, totaling 11,642, 9282, 9102, and 9678 patches, each 224 × 224 pixels. The CCP model outperformed state-of-the-art models UNet, Residual UNet (ResUNet), and Convolutional Neural Network Long Short-Term Memory (CNN-LSTM) with a Dice coefficient of 99.90%, an F1-score of 99.04%, a precision of 99.80%, and a recall of 99.76%. The learning process began with a learning rate of 0.01 and a decay rate of 0.8, and the SCNN achieved false negative and false positive rates of 0.04 and 0.05 for low-density nuclei at 400× and 40× magnification, respectively. In contrast, the Deep Convolutional Neural Network (DCNN) recorded rates of 0.02 and 0.01. For high-density patches, the SCNN model FN and FP rates of 0.0 and 0.08, while the DCNN reported 0.09 and 0.0. The proposed learning process with Smart Neural Networks (SNN) achieved high precision (77–99%), recall (75–99%), F1-score (75–99%), and an AUC of 86–100%. The combination of CCP and SNN improved accuracy over existing CNN models like ResNet50, VGG19, DenseNet109, DenseNet201, and VGG16. An ablation study showed a p-value of 0.00003 based on the AUC, highlighting the model’s potential to enhance automated breast cancer diagnosis and support clinical decision-making.

The World Health Organization (WHO) states that cancer accounts for 9.3 million deaths globally, making it a leading cause of mortality, as shown in Fig. 1. Around 19.6 million new cancer cases are reported annually and are listed in Table 1. The International Agency for Research on Cancer (IARC) predicts that cancer cases and deaths may double by 2040, with breast cancer cases potentially increasing by 40%. The increasing incidence of breast cancer underscores the need for early detection through screening technologies like digital mammography, though ultrasound remains cost-effective, and Magnetic Resonance Imaging (MRI), incorporated with microwave-based imaging systems using a cylindrical antenna with a meta-surface design. The expertise of skilled radiologists is essential for ensuring precise diagnostic outcomes. Meanwhile, histopathology plays a vital role in the diagnostic process, even though it faces various accuracy-related challenges. This intricate interplay between imaging and tissue analysis highlights the importance of both fields in achieving reliable patient diagnoses^1–19^.

**Fig. 1:**
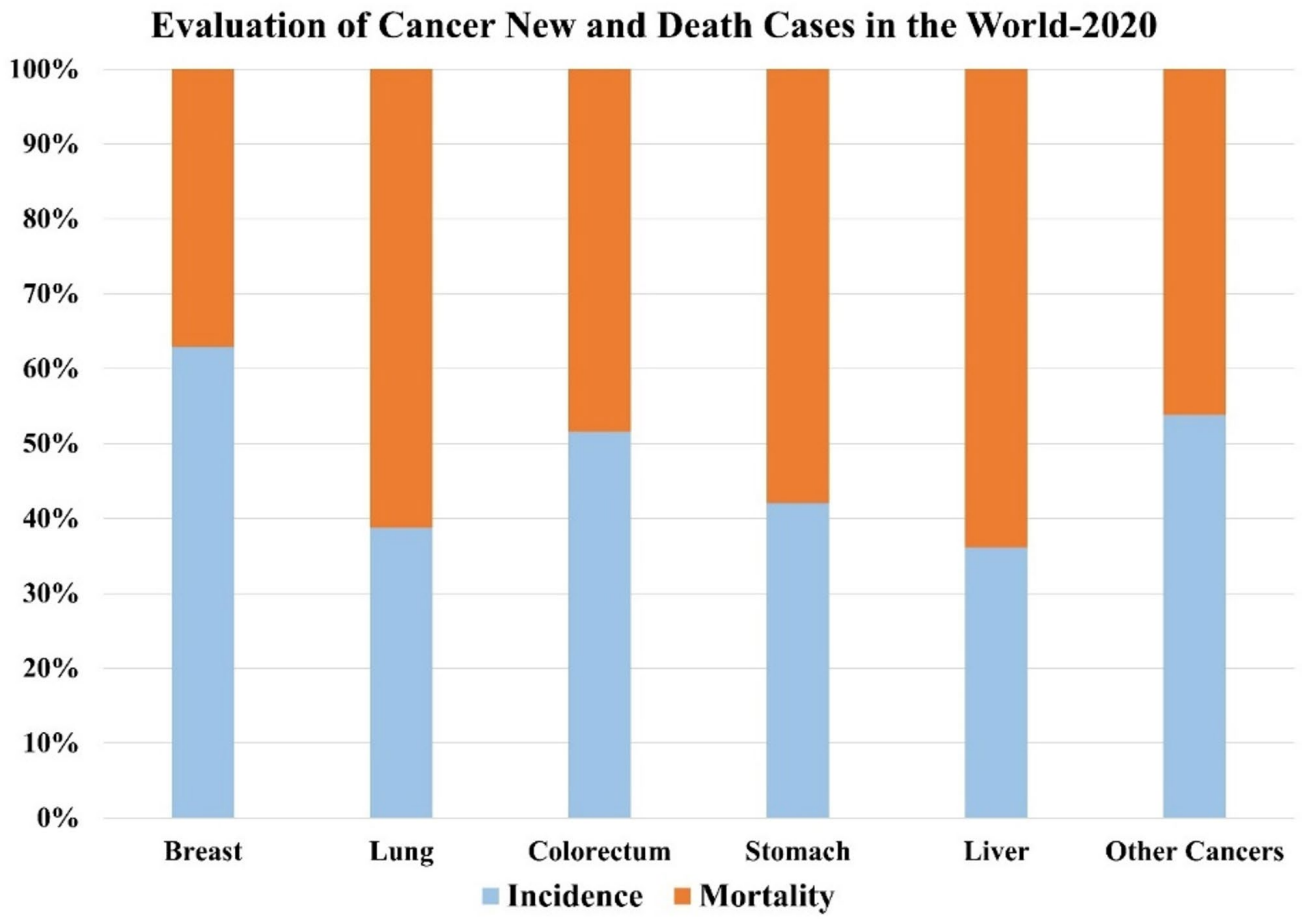
Comparison of 19.6 million new cancer cases and 9.3 million cancer-related deaths.

**Table 1:** The WHO 2020 report includes cancer statistics (in millions) for new cases and deaths over the past 5 years.

Cancer types	Incidence	Mortality	Prevalent
Breast	2,261,419	684,996	7,790,717
Lung	2,206,771	1,796,144	2,60,4791
Colorectum	1,931,590	935,173	5,253,335
Prostate	1,414,259	768,793	4,956,901
Stomach	1,089,103	3,932,768	1,805,968
Other cancer	8,879,843	3,932,768	24,433,023
Total	19,292,789	9,958,133	50,550,287

There are several effective methods for classifying breast cancer (BC). The Adam Golden Search Optimization-based Deep Convolutional Neural Network (AGSO-DCNN) efficiently extracts features and shapes to differentiate between non-tumor and tumor nuclei, employing Gaussian filters for noise removal and reducing time complexity. It utilizes shape features, Pyramid Histogram of Oriented Gradients (PHOG), and Local Vector Pattern (LVP). Convolutional Neural Networks (CNNs) have significantly advanced breast cancer classification through histopathology images^20^. Notably, a Twin CNN architecture has been developed to enhance multimodal feature extraction^21^, while another approach has integrated CNNs with ResNet34, VGG16, and AlexNet^22^, using Lipschitz-based image augmentation techniques. The early net model VGG11 and EfficientNet have accurately distinguished between benign and malignant Invasive Ductal Carcinoma (IDC) cases^23^. The proposed Deep Learning-based Enhanced Breast Invasive Ductal Carcinoma Model (DEBCM) utilizes CNN architecture with transfer learning and data augmentation to improve classification accuracy between cancerous and non-cancerous IDC tumors^24^. Nature-inspired metaheuristic algorithms have shown promise in improving medical image analysis. Inspired by geese migration, the Greylag Goose Optimization (GGO) algorithm demonstrated strong performance on benchmark datasets^25^. Likewise, a hybrid PSOBER optimizer enhanced a CNN-DBN model for oral cancer detection, achieving 97.35% accuracy. These works highlight the value of hybrid optimization in medical diagnostics^26–29^.

Building on this, our study targets breast cancer image classification and segmentation using UNet optimized deep learning techniques for improving accuracy and interpretation. Nuclei segmentation in histopathology images of breast cancer presents a myriad of challenges stemming from the inherent diversity of tissue samples. This complexity is exacerbated by multiscale features that demand sophisticated analysis at various resolutions. Additionally, variability in staining techniques can lead to significant differences in color and contrast, complicating the accurate identification of cell boundaries. Overlapping nuclei further intensify the segmentation difficulties, as they obscure clear delineation between individual cells. Coupled with potential image artifacts, these factors create a formidable landscape for effective nuclei segmentation, necessitating advanced computational approaches and robust algorithms for accurate analysis.

Prusty et al. address the challenges associated with staining inconsistencies in their work. They proposed a Structure-Preserving Color Normalization (SPCN) technique, which aims to standardize various stains and improve the performance of the ensemble U-Net model architecture. However, a limitation of the SPCN technique is that it does not account for nonlinear diverging datasets, and its normalization methods may suppress necessary intensity diagnostic features^30^. Khan et al. address the challenges of segmenting overlay nuclei, specifically the loss of spatial details that occurs when dealing with small, heterogeneous nuclei during down-sampling, often due to low image contrast. They propose a hybrid U-Net model integrated with Discrete Wavelet Transform (DWT) to tackle these challenges. These approaches enhanced feature extraction during both the encoding and decoding processes and incorporated spatial-channel attention, improving clustered nuclei separation. Additionally, this work addresses research gaps concerning model complexity and generalization^31^.

Chen et al. address the challenges associated with blurred boundaries and the unpredictable segmentation of small nuclei in input images. They proposed the Spatial Frequency Enhancement Network (SFE-Net) model to enhance both edge clarity and the consistency within nuclei. This work utilized a watershed post-processing algorithm to separate overlapping nuclei effectively; however, it is limited by its time complexity and is not robust for heavy artifact removal^32^.Ke et al. proposed a three-stage pipeline to address the challenges in artifact management. The stages include an Artifact Restoration (AR) classifier for removing artifact subtypes, RandStainNA for stain removal, and the Artifact Restoration Cycle Generative Adversarial Network (AR-Cycle GAN) for artifact restoration, which preserves the structure of the nuclei. However, this proposed work is limited in removing rare artifact types. It highlights a significant research gap in stain-preserving augmentation and restoration, particularly in the context of a fully automated pipeline^33^.

To address the challenges of the existing work, in this study, we proposed a hybrid model that combines the CCP and SMART CNN models for efficient nuclei detection and classification of breast cancer in histopathology images using the benchmark BreakHis dataset. The significant contributions of the work are summarized as follows.

- First, two proposed models are introduced, one for nuclei detection and segmentation, and another for the classification of BC histopathology images. The proposed CCP introduced the Deep U-Net SAM for nuclei detection and efficient segmentation. The nuclei density is calculated using microns per pixel as an efficient measure to locate the nuclei in each patch.
- Second, the state-of-the-art is to trigger the model to learn more efficiently the essential features from the complex structure of the histopathology images. The patches are clustered into Low Density (LD), Medium Density (MD), and High Density (HD) images. The proposed SNN introduces SCNN and SDNN for the classification of BC by taking the clustered patches as input and achieving outstanding performance in the classification results of benign and malignant samples.

**Fig. 2:**
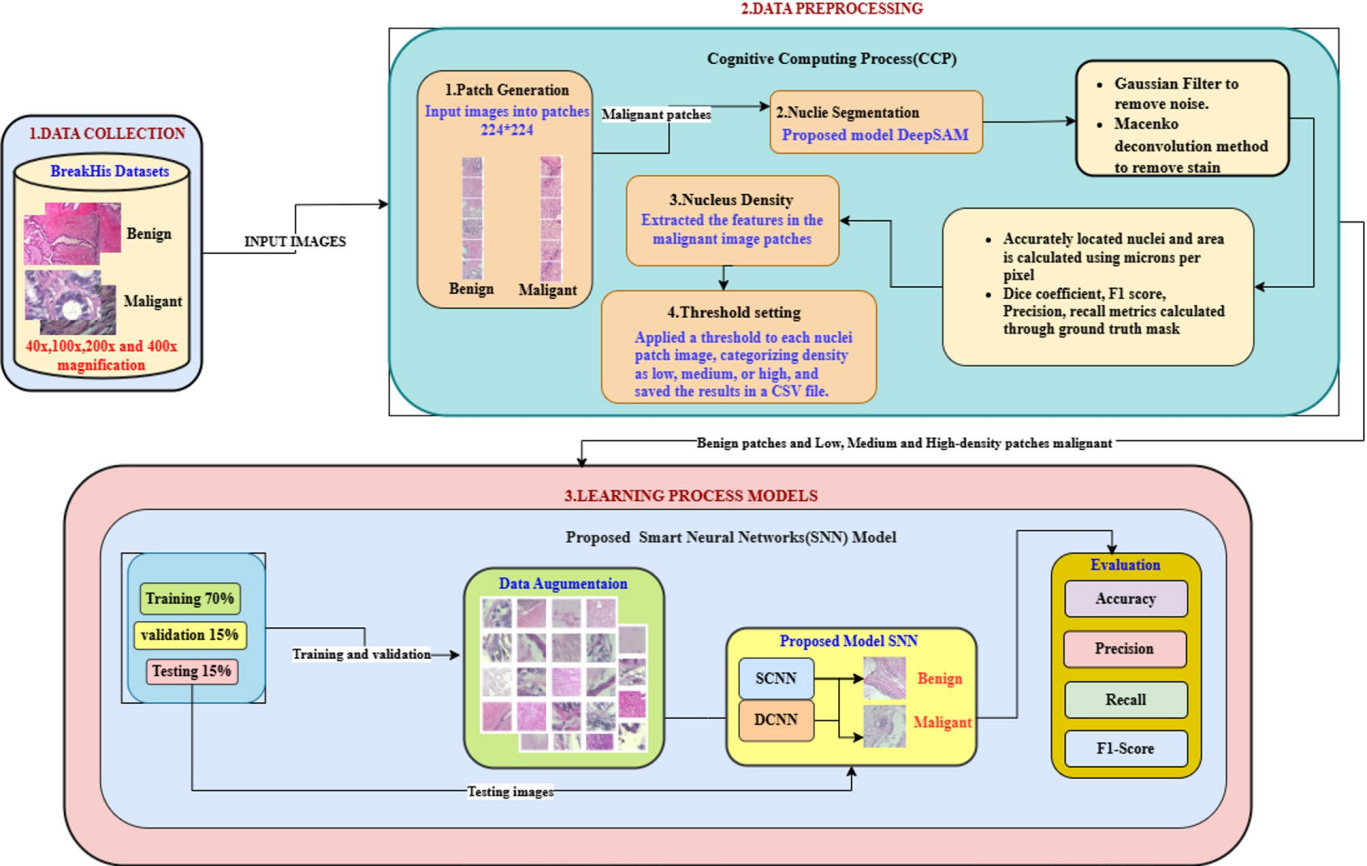
Workflow of the proposed architecture.

Figure 2 illustrates an overview of the proposed architecture. The main objective of this study is to efficiently train the model by providing effective inputs that allow it to learn and extract features from both benign and malignant samples. We collected breast cancer histopathology samples from the BreakHis dataset at various magnifications. These samples are processed using two different hybrid model pipelines: one that employs the CCP method and another that utilizes the Learning Process Intelligent Model.

In the first method, instead of resizing the input images, we convert them into patches of size 224 × 224 for both benign and malignant samples. This approach ensures the model learns all the relevant features from the input images. To accurately locate the nuclei, we introduce the Deep U-Net SAM model, which detects the boundaries of the nuclei and calculates their density for each patch using micron-per-pixel measurements and thresholding values. Each patch is then saved based on the nuclei counts, categorized as low, medium, or high density. Finally, SNN models are introduced to classify breast cancer images, distinguishing benign and malignant samples across low, medium, and high densities. This hybrid technique enables efficient learning of malignant images while minimizing the misclassification of benign images.

The article is structured to include the following sections in the “Materials and methods”. It discusses data collection and the relevant preprocessing techniques applied to input images from various methods (CCP) analysis. This includes patch generation, detecting nuclei using microns per pixel for segmentation, calculating their density, and separating them based on threshold values for Low Density (LD), Medium Density (MD), and High Density (HD). In the “Methods” section, the input data is trained and tested using the proposed Smart Neural Network (SNN) model to enhance the learning process methods. The “Results and discussion” section compares the proposed model with recent existing models, includes an ablation study, and identifies research gaps and suggestions for Future work in the “Conclusion”.

## Materials and methods

**Fig. 3:**
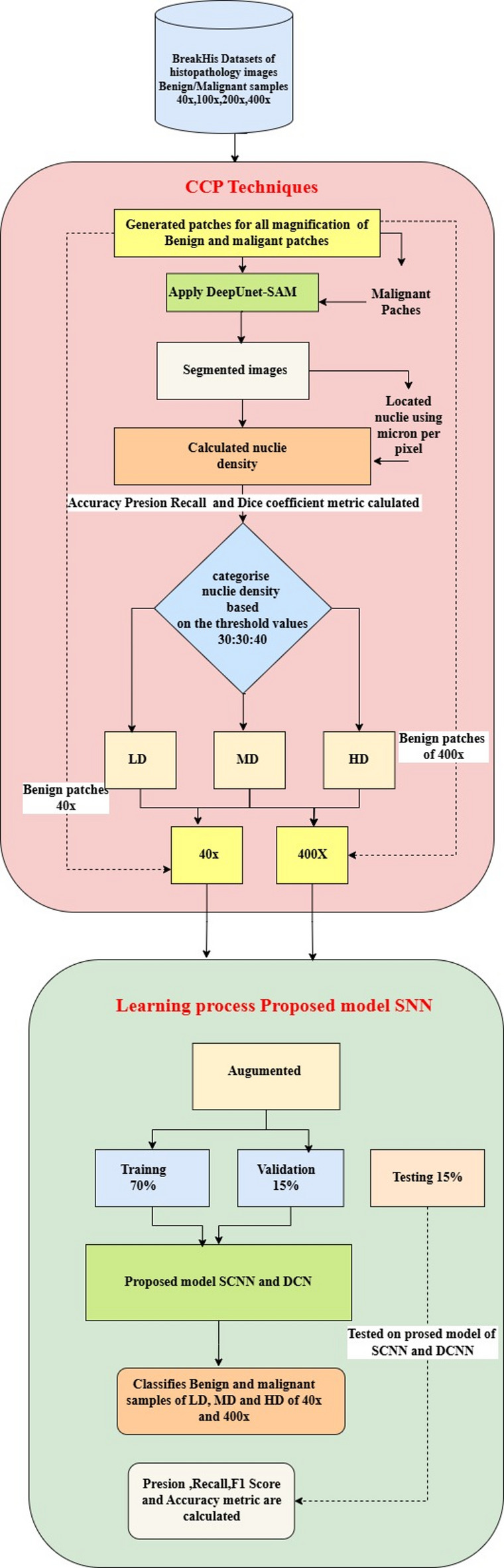
Workflow of the proposed method.

The main objective of the proposed work is to analyze the model’s learning process through pre-processing the images from the BreakHis dataset at different magnifications (40×, 100×, 200×, and 400×). The Cognitive Computing Process (CCP) is employed to accurately locate and extract nuclei from the images, focusing on 40× and 400× magnifications for deeper insights. Algorithm for the proposed work is presented below, and Fig. 3 illustrates the workflow of the proposed methodology.

### Algorithm of the proposed work

Step 1: Collected breast cancer histopathology images from the BreakHis dataset at 40×, 100×, 200×, and 400× magnifications.

Step 2: Read images containing benign and malignant samples at all magnifications.

Step 3: The CCP method started, to retain all information from the images, the whole image 700*460 is converted into patches of size 224 × 224.

Step 4: Deep U-Net SAM is used to locate the nuclei accurately using microns per pixel, and the density is calculated for the malignant image.

Step 5: Threshold is applied for the nuclei density as a 30:40:40 ratio, dividing into LD, MD, and HD, and assessed performance metrics across magnifications.

Step 6: Benign patches are incorporated into a folder of LD, MD, and HD for learning.

Step 7: To enhance the learning process of the SNN classification model introduced.

Step 8: SNN model of DCNN and SCNN trained, validated, and tested on various density patches LD, MD, and HD images at 40× and 400×, and classified benign and malignant samples in various densities.

Step 9: The SNN model proves that the binary classification result is higher in 40× magnification and 400×.

### Data collections

**Fig. 4:**
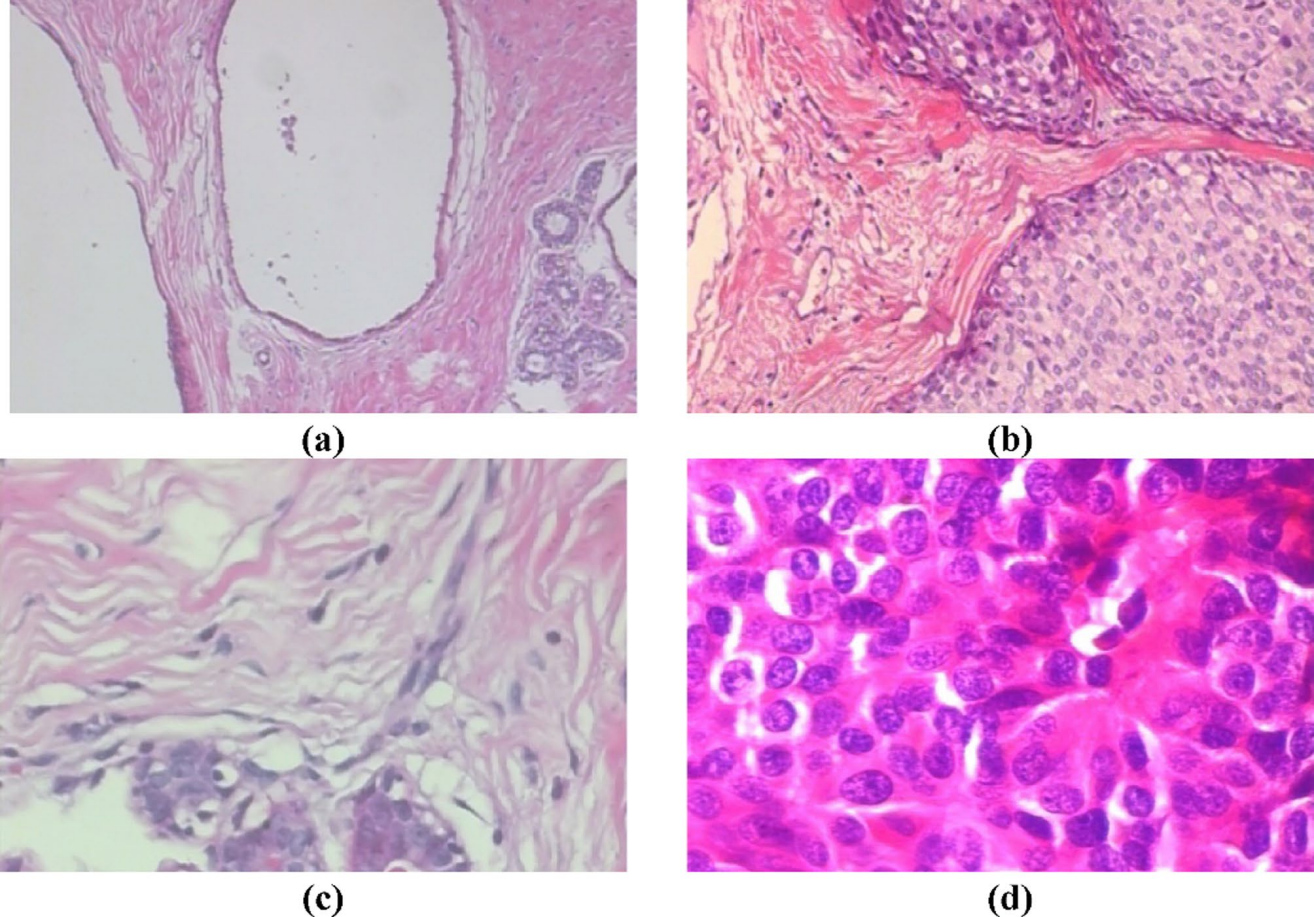
Histopathology images of benign and malignant nuclei at different magnifications: (**a**) 40×, (**b**) 100×, (**c**) 200×, and (**d**) 400×.

The BreakHis dataset is a significant contribution from the Vision Robotics and Imaging Lab at the Federal University of Paraná in Brazil. The images, measuring 700 × 460 pixels with three RGB channels and 8-bit depth, are stored in PNG format to ensure high quality^34^. The dataset details are shown in Table 2, and sample images are in Fig. 4.

**Table 2:** Represents the breakhis histopathology images dataset at various magnification: 40×, 100×, 200×, and 400×.

Class	Sub-types	Number of patients	Magnification				Total
			40×	100×	200×	400×	
Benign	Adenosis	4	114	113	111	106	444
	Fibroadenoma	10	253	260	264	237	1014
	Tubular adenoma	3	109	121	108	115	453
	Phyllodes	7	149	150	140	130	569
Total	Magnification	24	625	644	623	588	2480
Malignant	Ductal	38	864	903	896	788	3451
	Lobular	5	156	170	163	137	626
	Mucinous	9	205	222	196	169	792
	Papillary	6	145	142	253	138	678
Total	Magnification	58	1370	1437	1508	1232	5547

## Data preprocessing

### Cognitive Computing Process (CCP)

CCP allows the model to focus on fine details and critical patterns within the nuclei, leading to more precise learning outcomes. The model is better equipped to accurately diagnose malignancies by honing in on these intricate features. As a result, the CCP significantly improves feature extraction while effectively minimizing the time and computational complexity in the classification process. Unlike traditional methods that depend on images across all magnifications, CCP concentrates explicitly on processing high-magnification (400×) to low-magnification (40×) images. Focusing on these magnification levels, the CCP better analyzed cellular and tissue characteristics, crucial for early breast cancer diagnosis. The CCP enhances the accuracy and effectiveness of the diagnostic process, paving the way for improved outcomes in breast cancer detection.

**Fig. 5:**
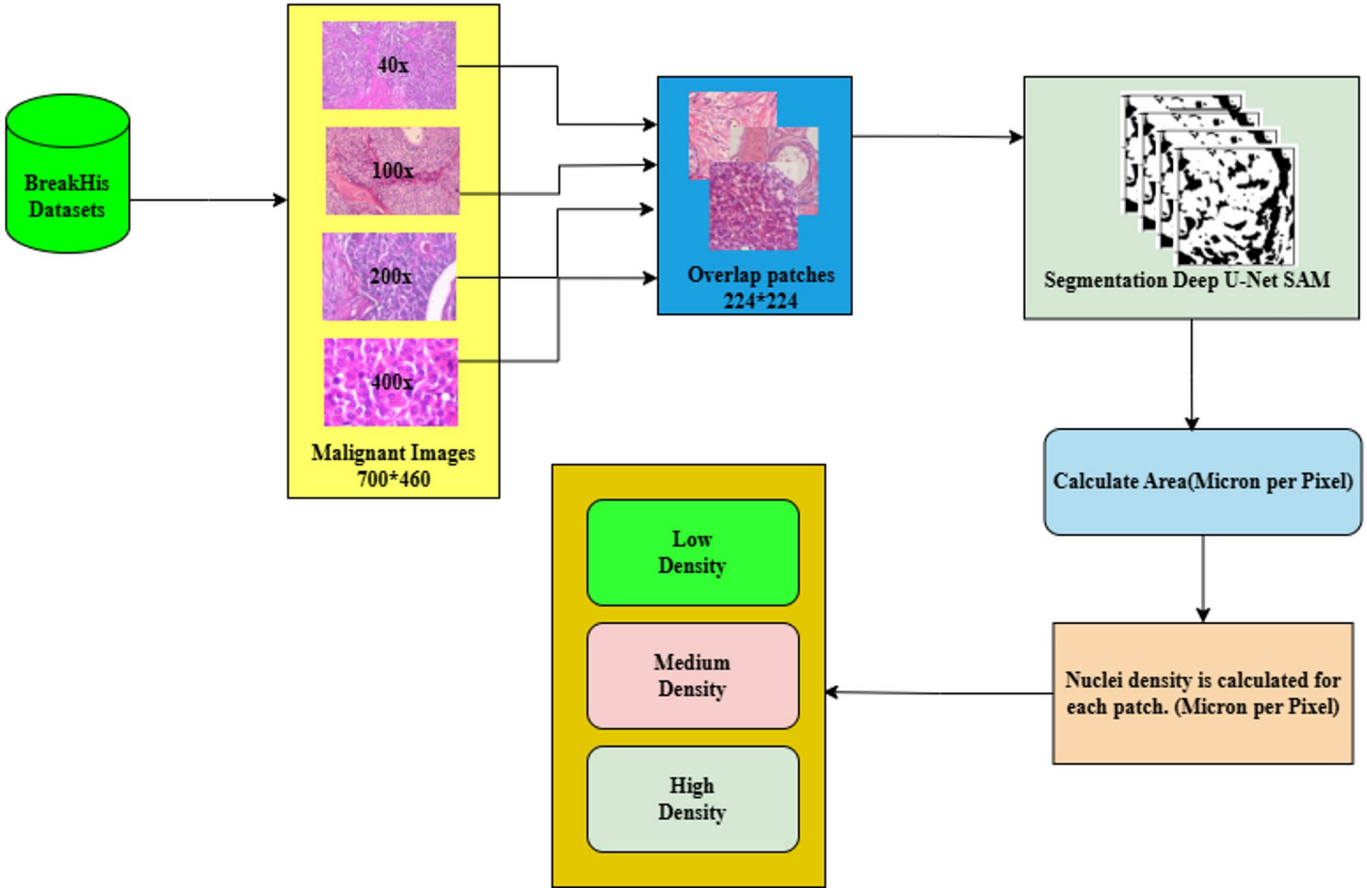
CCP architecture.

This proposed work aims to boost the model’s productivity through an efficient and effective learning process by providing high-quality input data. This enhances the model’s ability to diagnose the early stages of breast cancer effectively despite low-magnification images. Figure 5 demonstrates the CCP architecture, and the following approaches are given below.

- Patch generation: Splitting the whole image into patches ensures that the image retains the original features and is compact to our model. To retain the tissue boundary of the nuclei, overlapping patches are generated, and the patches are limited until all the pixels are covered in alternate patches.
- Nuclei segmentation: Segmenting nuclei from input malignant image patches using the advanced Deep U-Net Spatial Attention Model (SAM) accurately identifies and outlines the nuclei within each patch.
- Nucleus density calculation: We meticulously calculated the density of nuclei for the unique malignant patch using micron-per-pixel values, which is crucial for understanding the progression and severity of the malignancy.
- Threshold setting: The patches are classified into low, medium, and high-density categories based on the density of malignant cell nuclei. These clusters are crucial for accurately categorizing the patches according to their characteristics. This method effectively trains our model, leading to a more precise and informed analysis of the early stages of breast cancer.

#### Patch generation

**Fig. 6:**
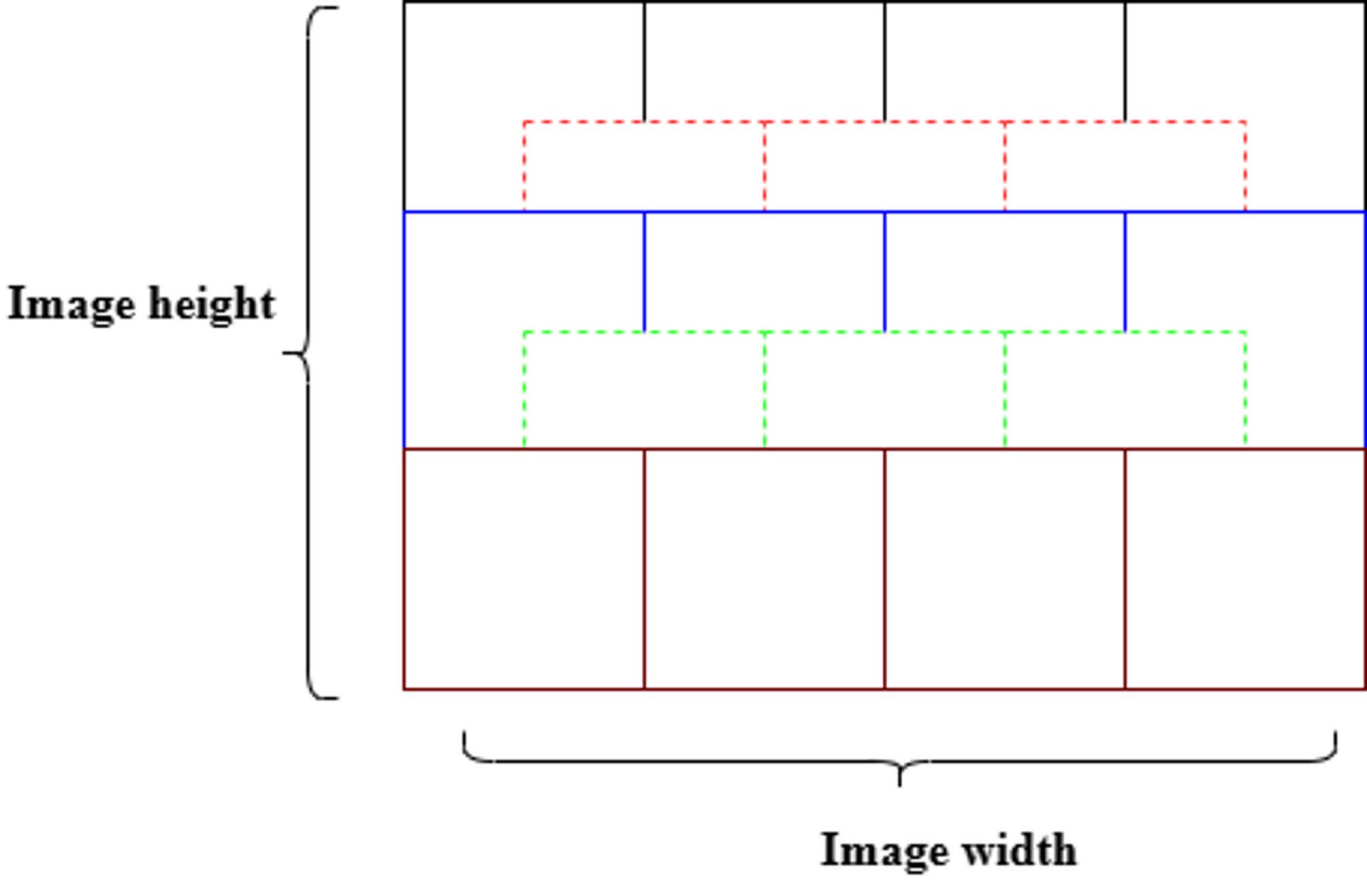
Outline of image patches sized 224 × 224 pixels.

The datasets are split based on four specific magnification levels: 40×, 100×, 200×, and 400×. Each class encompasses benign and malignant samples, with every image comprising 700 × 460 pixels. Instead of resizing these images, which leads to a loss of detail, overlap patches measuring 224 × 224 pixels are meticulously extracted at each designated magnification level to regain the boundary of the nuclei and limit the patches until all the pixels are covered, as outlined in Table 3 and shown in Fig. 6.

**Table 3:** Histopathology image samples used in this proposed study.

Class	Whole Images in 700 × 460-pixel size				Patched Images in a 224 × 224-pixel size			
	40×	100×	200×	400×	40×	100×	200×	400×
Benign	625	644	623	588	6198	3864	3756	3528
Malignant	924	903	896	1025	5544	5418	5376	6150
Total	1551	1547	1522	1513	11,642	9282	9132	9678

#### Nuclei segmentation

**Fig. 7:**
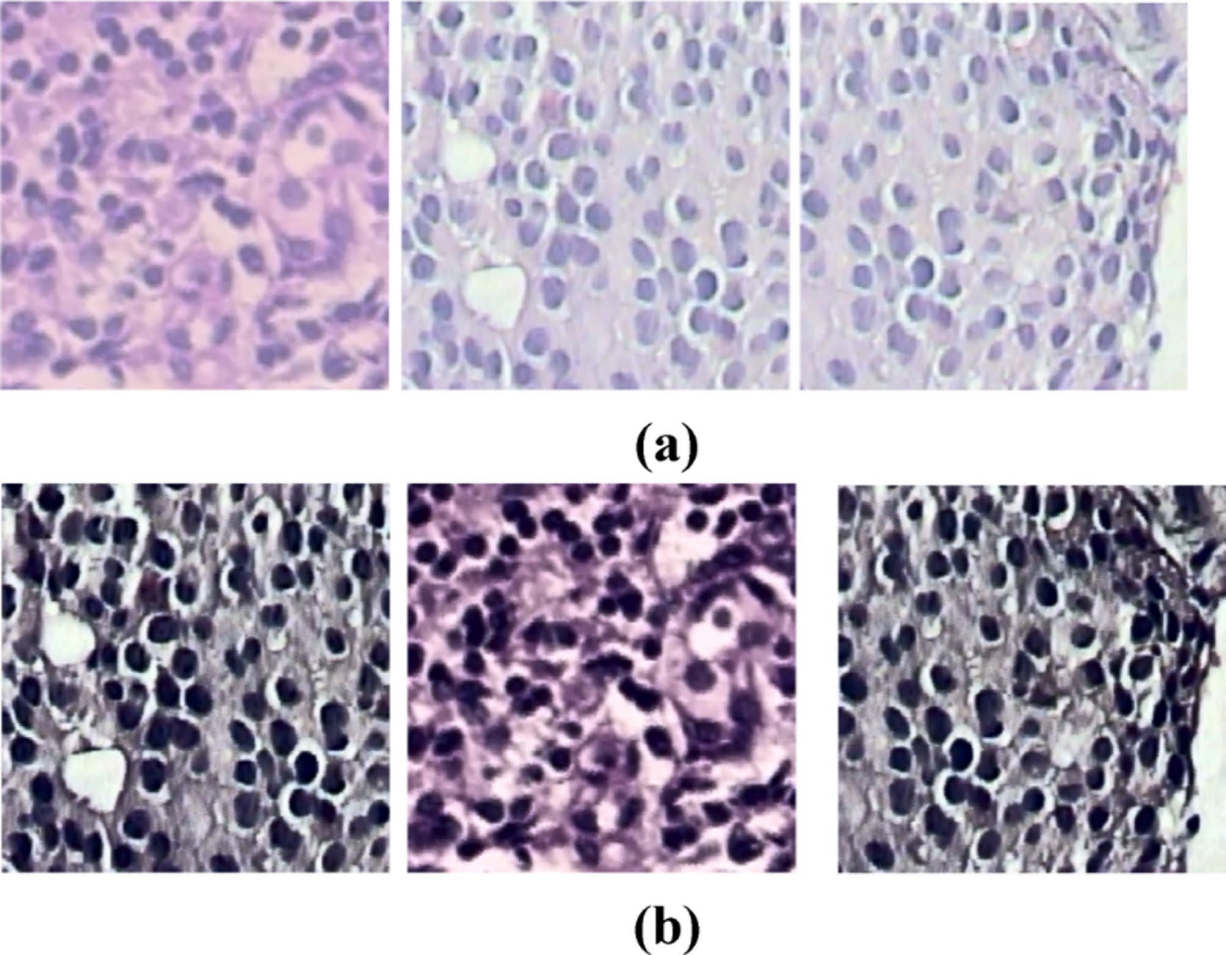
Deconvolution method applied to histopathology images for remove stain (**a**) Original Image (**b**) stain-removed image.

Detecting overlapped nuclei in histopathology images is challenging due to the hidden features within these images. The U-Net model has become a key framework for medical image segmentation, leading to various adaptations to enhance performance^35–40^. This study employs a Gaussian filter to reduce noise in input patches, resulting in cleaner data for better analysis. Additionally, the deconvolution method is used to remove Hematoxylin-Eosin(H&E) staining, effectively revealing the underlying cellular architecture, and sample stain-removed images are shown in Fig. 7. Our model segments overlapped malignant image patches at different magnifications (40×, 100×, 200×, and 400×) using the Deep U-Net Spatial Attention Model (Deep U-Net SAM), which excels at locating, detecting, and segmenting nuclei. The encoder’s deep feature maps and the decoder’s attention mechanism allow for precise identification and delineation of nuclei in each patch.

**Fig. 8:**
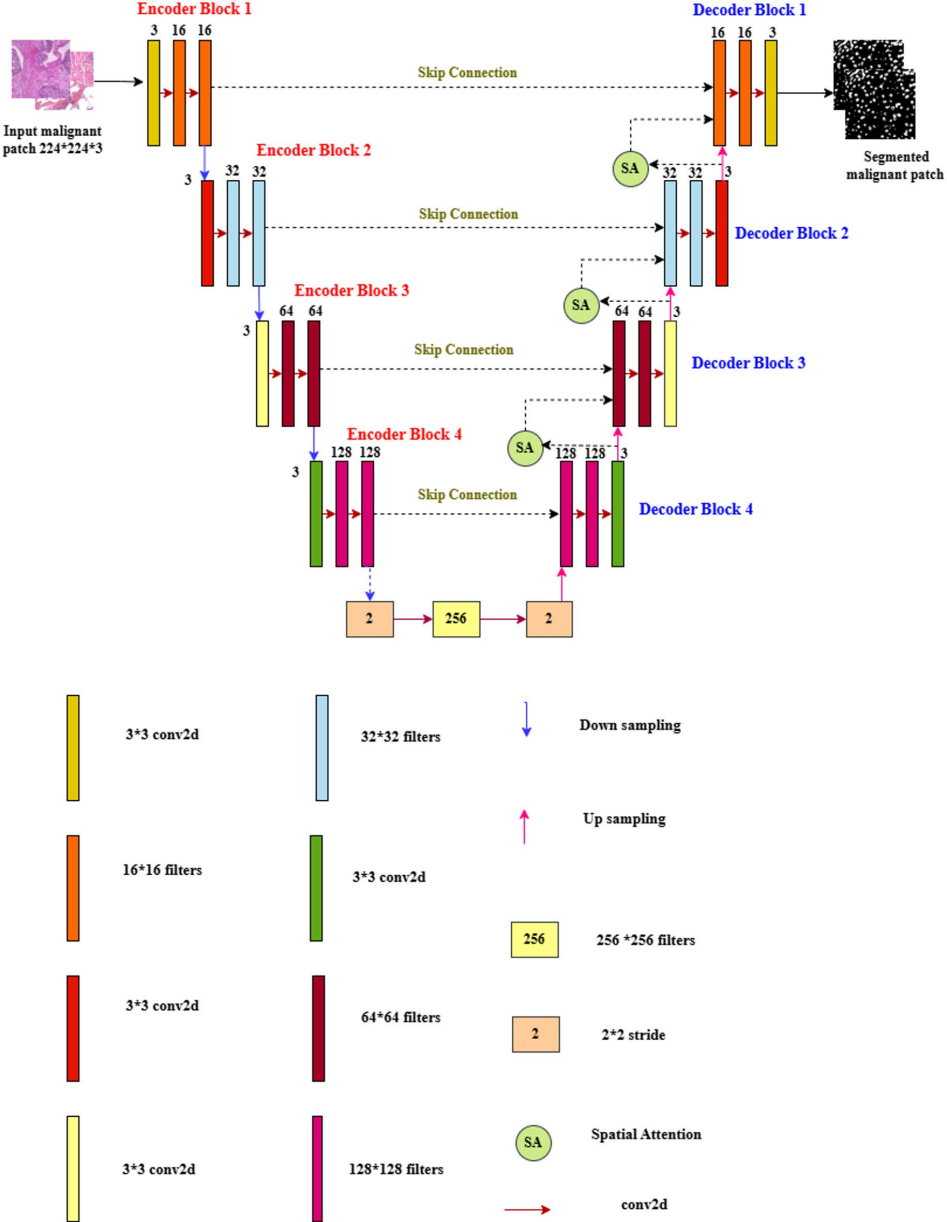
Proposed deep U-Net model with spatial attention mechanism (SAM).

Figure 8 demonstrates the proposed Deep U-Net SAM model, which is characterized by a sophisticated architecture that comprises both encoder and decoder sections, working in tandem to process and reconstruct data effectively. Within the encoder, a series of progressive down-sampling operations is meticulously executed, while the decoder employs corresponding up-sampling techniques, both accomplished through the application of convolutional operations. This architecture is built upon a foundation of  × 3 convolutional layers, utilizing a range of filter sizes specifically, 16 × 16, 32 × 32, 64 × 64, 128 × 128, and 256 × 256, ensuring an optimal balance between feature extraction and computational efficiency. Each convolutional layer is fitted with a stride of 2 × 2, which facilitates the gradual reduction of spatial dimensions within the encoder. Moreover, the model incorporates four strategically placed max-pooling layers, each with a pooling size of 3 × 3, further enhancing the dimensionality reduction process while retaining critical features for subsequent stages.

A noteworthy feature of the decoder block is that the layers are integrated with a spatial attention mechanism (SAM), allowing the model to focus on important regions of the data and improving the interpretability and accuracy of the output. To reduce nonlinearity in the model, the Rectified Linear Unit (ReLU) activation function is employed throughout the architecture, ensuring that a wide variety of functions can be learned from the data. Finally, the model culminates in a classification layer that utilizes a sigmoid activation function, effectively converting the output features into probabilities for binary classification tasks. This thoughtful design allows the Deep U-Net SAM model to excel in complex data processing scenarios. Accurate detection of nuclei in histopathology images is essential for successful diagnostic and research applications.

**Fig. 9:**
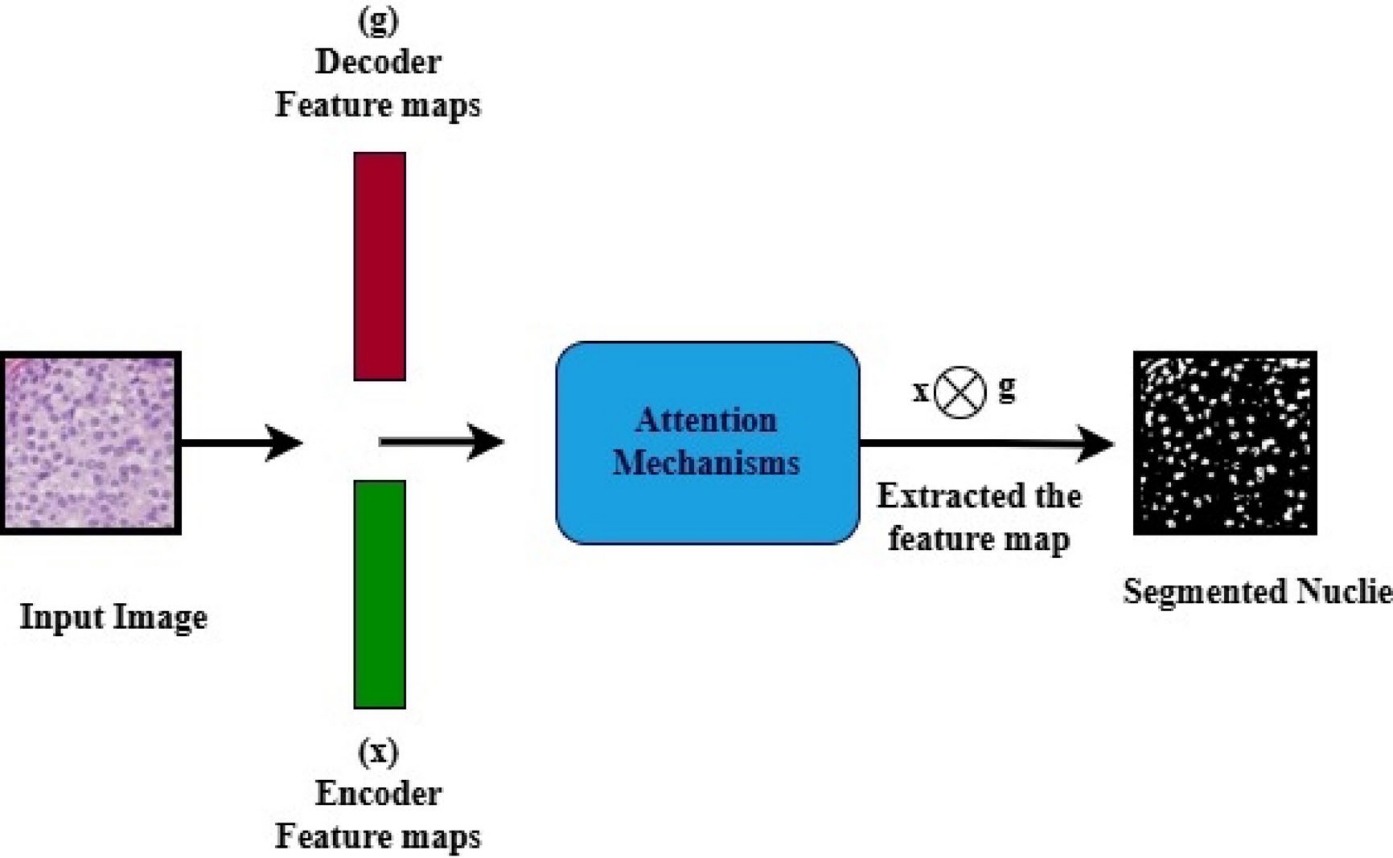
Architecture of the spatial attention mechanisms (SAM).

Figure 9 illustrates Spatial Attention Mechanisms (SAM). In the SAM model, the malignant images are fed into an encoder that extracts spatial features from both the encoder and decoder. The decoder aids in directing the attention mechanisms to focus on the noise and nuclei regions. The attention gates learned to suppress irrelevant background noise and enhanced discriminative features for precision. The attention module multiplies the features from the encoder by attention weights derived from the inputs of both the encoder and decoder, which enhances significant features while diminishing the background. These results improved the efficiency of nuclie segmentation.

The Deep U-Net SAM model is trained over 50 epochs in this approach to ensure it effectively captures the complex features inherent in the input image patches. The specific parameter settings utilized for the Deep U-Net SAM model are meticulously outlined in the accompanying Table 4, providing insight into our methodological choices. Micron-per-pixel measurements are employed to precisely locate the nuclei, significantly improving their spatial identification accuracy. This process involves several well-defined steps, outlined below, to ensure that the model delivers reliable and consistent results in identifying cellular structures within the histopathological images.

**Table 4:** Parameter settings of the deep U-Net SAM model.

Parameters	Configurations of deep U-Net SAM model
Activation function	Sigmoid
Learning rate	0.0001
epochs	50
Batch size	16
optimizer	Adam
Loss function	Binary cross-entropy

#### Nuclei density calculation

These steps outline the process for calculating nuclei density.

- Microscope setup.
- Microns per pixel calculations.
- Nuclei detection.
- Estimation of nuclei area.
- Compute nuclei density.

#### Microscope setup

The BreakHis image datasets were captured using the Olympus BX-50 microscope with a 3.3× relay lens and a Samsung SCC-131AN digital camera. This camera features a 1/3-inch Sony HAD CCD with a pixel size of 6.5 μm × 6.25 μm and a resolution of 752 × 582 pixels, producing 24-bit RGB images. It supports objective lenses with magnifications of 40×, 100×, 200×, and 400×. Original images often have black borders and text annotations, which are cropped for improved quality. Edited images are saved as 700 × 460-pixel PNG files in three-channel RGB format, maintaining visual integrity without artifacts. Pixel size is calculated by dividing the camera’s physical pixel size by the relay and objective lens magnifications.

#### Microns per pixel calculations

This work uses microns per pixel measurements to analyze cell architecture in histopathology images. Microns are essential in microscopy for determining object sizes and cell structures in whole-slide images, including those in the BreakHis datasets. The micron-per-pixel value represents the physical size of each pixel, calculated from metadata of the datasets referenced in^41–43^. The camera sensor has a consistent pixel size of 6.5 μm across various magnifications (40×, 100×, 200×, and 400×) and lenses (4×, 10×, 20×, and 400×). Magnification depends on the lens, which focuses light for clear imaging, allowing for accurate size representation.

Table 5 shows the micron pixel values for different magnifications using Eq. (1).

1 $$\:{mpp}_{\:\:\:\:}-\:\frac{pps}{{m}_{\:\:\:\:\:}}{\upmu\:}\text{m}\:$$

Let, µm, micron meter, $\:{mpp}_{\:\:\:\:\:}$, micron per pixel, $\:\:\:\:\:\:\:{pps}_{\:\:\:\:}$, physical pixel size of the lens, $\:{m}_{\:\:\:\:\:}$ magnification

**Table 5:** Micron per pixel values at different magnification levels.

Visual magnification	$\:{m}{p}{p}\:(\upmu \text{m})$
40×	0.1625
100×	0.065
200×	0.0325
400×	0.01625

#### Nuclei detection

Calculating nuclei density within specific tissue areas is crucial for analyzing cellular composition. This involves measuring microns per pixel along the x and y axes using images at various magnifications (40×, 100×, 200×, and 400×). Higher magnification provides detailed insights into tissue architecture. Nuclei density is determined by the number of nuclei per unit area, converting pixel area into square units. The micron-per-pixel values for the x and y axes are calculated using Eqs. (2) and (3), leading to accurate measurements of nuclei area.

Table 6 illustrates constant values for different BreakHis dataset magnifications. These values remain consistent since the sensor pixel size and magnification factor do not change, ensuring uniform pixel dimensions and quality images.

2 $$\:{mpp}_{x}\:\:=\:\:\frac{pps}{m\:\:}{\upmu\:}\text{m}$$

3 $$\:{mpp}_{y}\:\:\:=\:\:\:\frac{pps}{m\:\:}{\upmu\:}\text{m}$$

where, $\:{mpp}_{x}$ is the micron per pixel respective to the x-axis, $\:{mpp}_{y}$ is the micron per pixel respective to the y-axis.

**Table 6:** Whole images micron per pixel values with respect to the x and y axis.

Visual magnification	Micron per pixel (µm)	
	$\:{{m}{p}{p}}_{{x}}$	$\:{{m}{p}{p}}_{{y}}$
40×	0.1625	0.1625
100×	0.065	0.065
200×	0.0325	0.0325
400×	0.01625	0.01625

#### Estimation of nuclei area

The calculation area of the nuclei is calculated using the following procedures, which are given in detail below.

- Read the height and width of the nuclei in millimeters (mm).
- To accurately locate the nuclei, the height and width of the nuclei are calculated to $\:{\upmu\:}\text{m}$ using Eqs. (4) and (5).

4 $$\:{w}_{x}-{w}_{p}\times\:{mpp}_{x}\left({\upmu\:}\text{m}\right)$$

5 $$\:{h}_{y}-{h}_{p}\times\:{mpp}_{y}\left({\upmu\:}\text{m}\right)$$

Equation (6) calculates the nuclei’s area.

6 $$\:{a}_{n}-{\:\:\:w}_{x}\times\:{h}_{y}\left({{\upmu\:}\text{m}}^{2}\right)$$

where, $\:{w}_{x}$ is the Width in micron meter, $\:{h}_{y}$ is the Height in micron meter, $\:{w}_{p}$ is the width (mm), $\:{h}_{p}$ is the height (mm), $\:{a}_{n}$ is the area of nuclei.

#### Calculate nuclei density

Table 7 shows nuclei values of unique malignant patches at various magnifications (40×, 100×, 200×, and 400×) along with the corresponding nuclei density. Finally, nuclei density is calculated for unique malignant patches by converting these values using Eqs. (7) and (8).

7 $$\:{a}_{mms}\:-\:\frac{{a}_{n}}{{10}^{-6}}\:\left({\text{m}\text{m}}^{2}\right)$$

8 $$\:{a}_{d}\:-\:\:\frac{n}{{a}_{mms}\:\:}{(\text{m}\text{m}}^{2})$$

where, $\:{a}_{mms}$ is the area of nuclei into millimetre square, $\:{a}_{d}$ is the nuclei density, $\:n$ is the total number of nuclei present segmentation region.

**Table 7:** Area and its conversion to square micrometres $\:\left({{\upmu\:}{m}}^{2}\right)$.

Magnification	$\:{{w}}_{{x}}$($\:{\upmu\:}{m})$	$\:{{h}}_{{y}}$($\:{\upmu\:}{m})$	$\:{{a}}_{{n}\:\left({{\upmu\:}{m}}^{2}\right)}$	$\:{{a}}_{{m}{m}{s}}$ ( $\:{{m}{m}}^{2})$
40×	36.4	36.4	1324.96	0.00132496
100×	14.6	14.6	213.16	0.00021316
200×	7.28	7.28	52.998	0.000052998
400×	3.64	3.64	13.25	0.00001325

#### Threshold settings

The nuclei density for each patch is calculated to determine its maximum density value. The malignant patches are then categorized based on this maximum nucleus density, using a threshold and a ratio of 30:30:40 for low, medium, and high density across different magnifications (40×, 100×, 200×, and 400×). Table 8 mentioned the total number of malignant density images along with sample images.

**Table 8:**
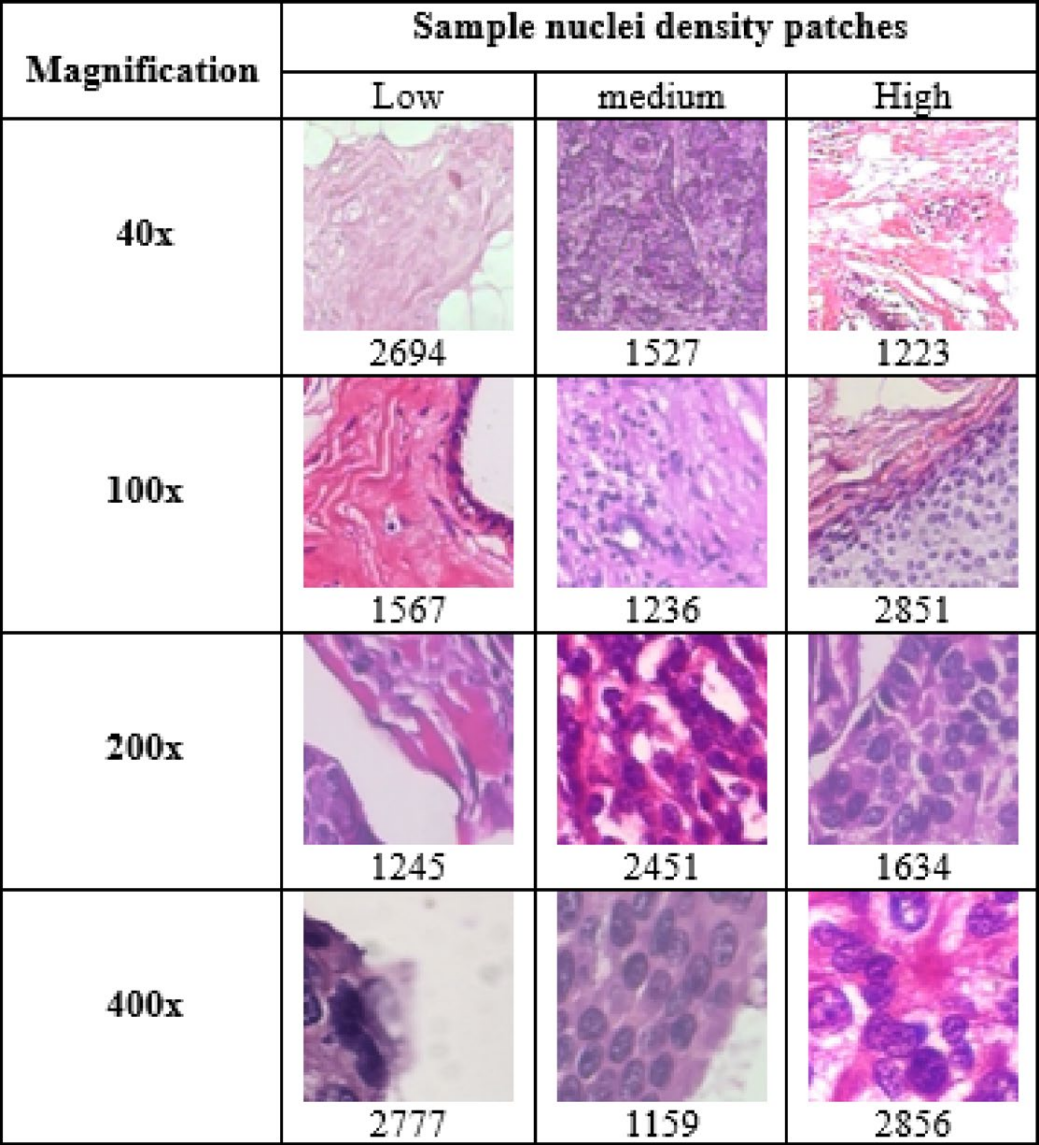
Samples of malignant nuclei density images (low, medium, and high) with the corresponding patch counts.

### Dataset split

**Fig. 10:**
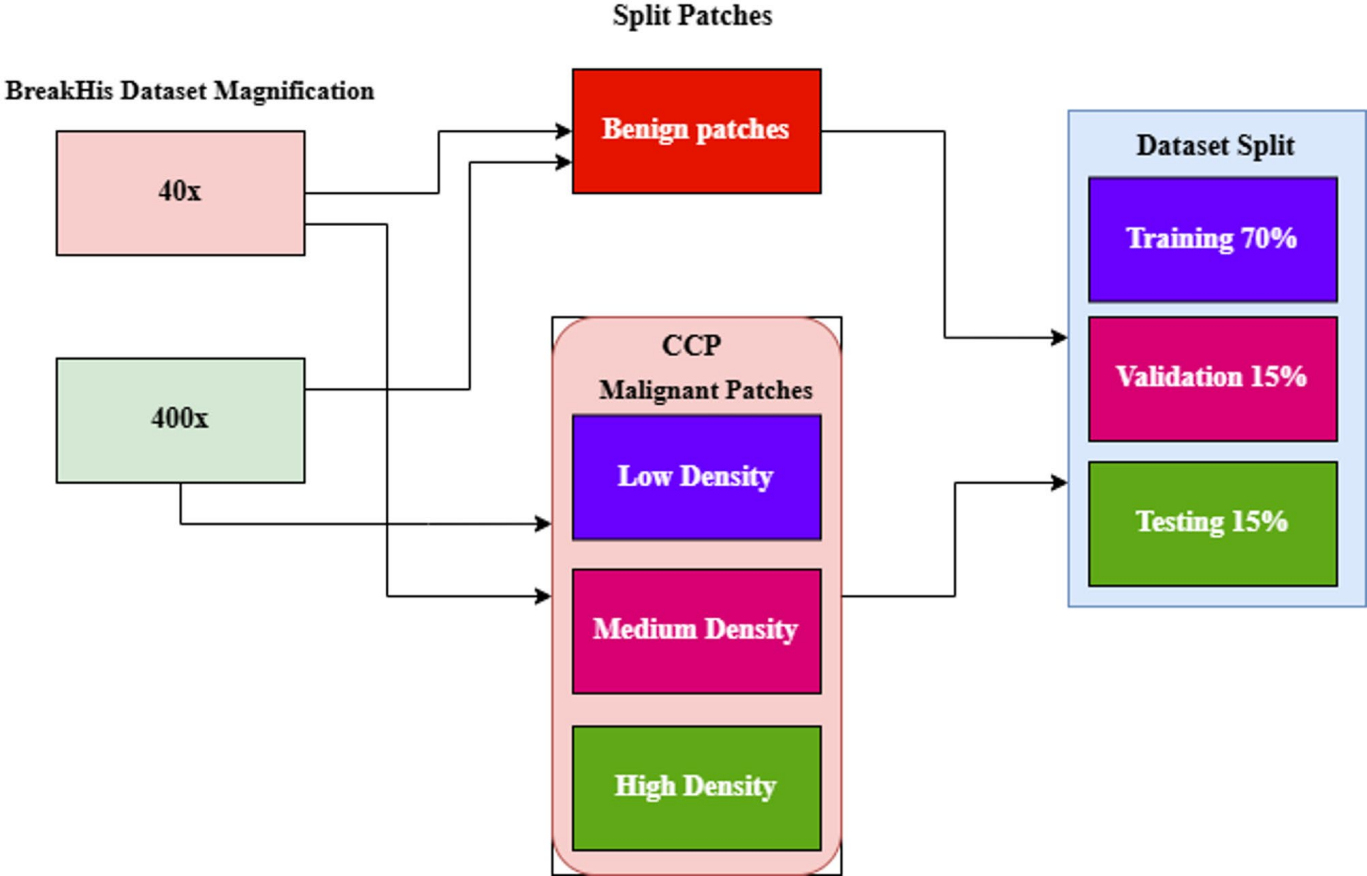
Datasets splitting techniques.

In this study, the magnifications 40× and 400× are used to highlight the model’s learning process. The malignant patches are classified into three categories based on their nuclei density: LD, MD, and HD. Figure 10 shows the dataset splits methods, and values are represented in Tables 9 and 10. The benign patches of 40× and 400× magnification are split as overlapping patches without measuring nuclei density. Various augmentation methods, including rescaling, zooming, shearing, rotating, and flipping, are applied to patched images using the ImageDataGenerator function with a batch size of 32.

**Table 9:** Dataset split of benign and malignant density patches at 40× magnification.

Patches	40x					
	Benign	Malignant-LD	Benign	Malignant-MD	Benign	Malignant-HD
Training	1963	1955	1291	1068	1083	856
Validation	278	230	278	230	233	184
Testing	420	419	276	229	232	183
Total	2651	2694	1845	1527	1547	1223

**Table 10:** Dataset split of benign and malignant density patches at 400× magnification.

Patches	400×					
	Benign	Malignant-LD	Benign	Malignant-MD	Benign	Malignant-HD
Training	2154	2136	819	810	2011	1999
Validation	324	321	175	176	432	429
Testing	323	320	175	173	431	428
Total	2801	2777	1269	1159	2874	2856

### SNN models

**Fig. 11:**
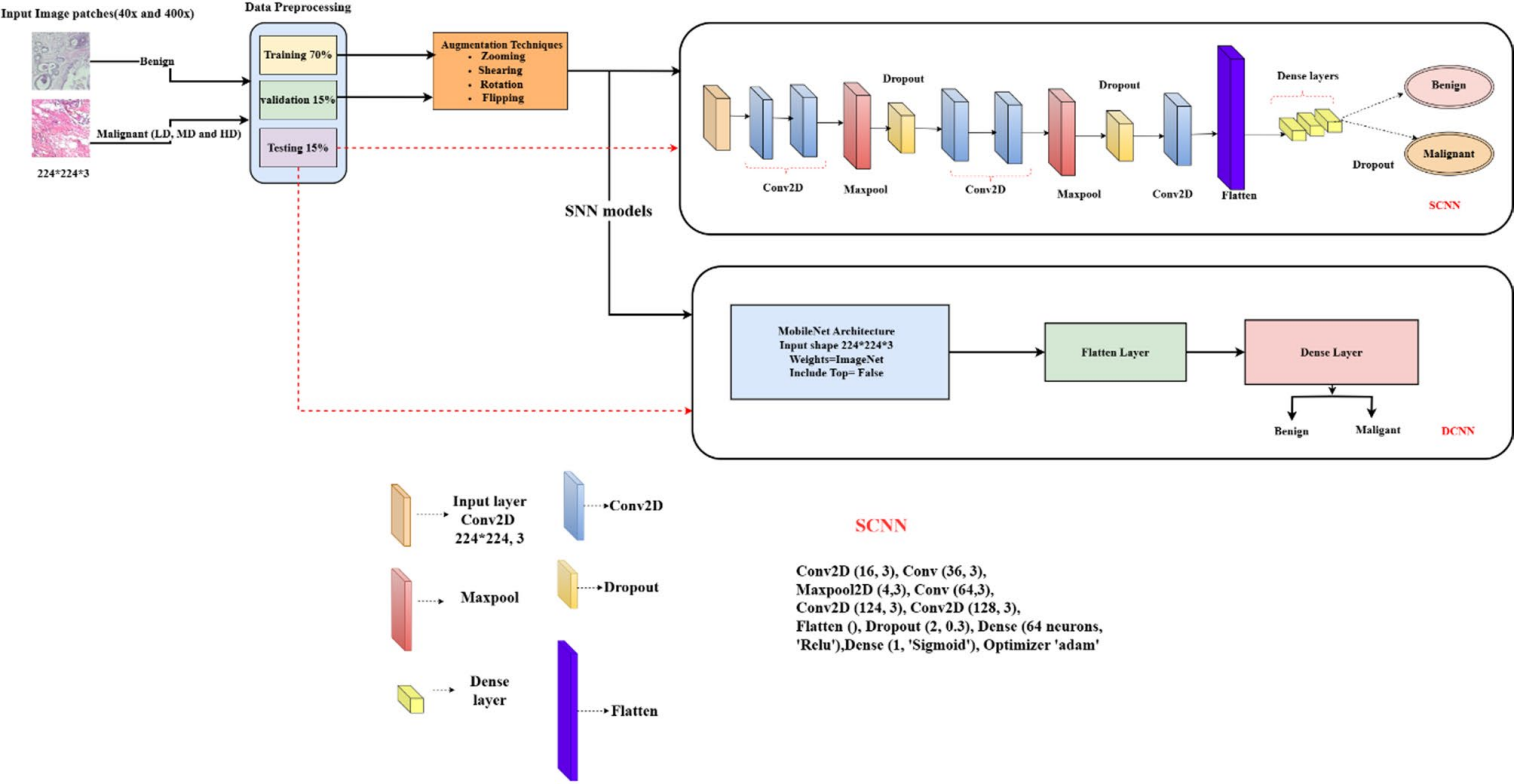
Architecture of the proposed model.

This methodology aims to enhance the learning rate and improve the efficiency of the learning process. User-defined thresholds calculate the nuclei density in malignant breast tissue histopathology images, classifying it into low, medium, and high levels. The model demonstrates more effective learning on different magnification images to offer a border view of the tissue. Figure 11 illustrates the proposed model architecture.

### SCNN

To achieve its goals, the CNN model is configured with specialized layers for efficient feature extraction. These layers are crucial for processing complex image data, enabling the model to recognize significant visual patterns that distinguish various nuclei densities. Tailored to detect intricate details, the SCNN architecture enhances classification accuracy, especially with 40x and 400x magnification images. This strategic design is essential for extracting relevant features and ensuring an effective learning process in understanding pathological features in breast tissue samples.

The model includes six 2D convolutional layers with filter sizes of 16, 32, 64, 128, and 256 to extract features from images, using a kernel size of (3, 3). Three 2D max-pooling layers of size (2, 2) reduce feature dimensions. To prevent overfitting, the model incorporates two batch normalization layers and three dropout layers with a retention rate of 30%. Features are flattened into a one-dimensional array and passed to the final classification layer, which consists of two dense layers using the ReLU activation function. However, this approach did not achieve the desired accuracy, prompting the introduction of a new learning strategy model called DCNN.

### DCNN

Transfer learning is an effective technique for training the initial layers of deep convolutional neural networks (DCNNs) to recognize image patterns. This research uses pretrained MobileNet networks, trained on the diverse ImageNet dataset with over 20,000 categories, allowing these models to learn rich feature representations that we refine for breast cancer classification^44^. DCNN model enhances the learning rate using the MobileNet model’s separable convolution architecture for improved feature extraction and model accuracy. The key components include:

- Input layer: Processes incoming data.
- Depthwise convolutional layers: Fourteen layers with filters (32, 64, 128, 256, 512, 1024), a kernel size of (3, 3), and stride (2, 2) for intricate feature extraction.
- Pointwise convolutional layers: Thirteen layers with a kernel size of (1, 1) refine features.
- Batch normalization layers: Twenty-six layers for stabilization and speed.
- ReLU activation layers: Twenty-seven layers for non-linearity and complex pattern capture.
- Zero padding layers: Four layers to maintain spatial dimensions and reduce overfitting.
- Flatten layer: Converts output to one-dimensional for classification.
- Output dense layer: One layer with two neurons for multiclass classification.

DCNN significantly improves learning rate and accuracy over SCNN, making it a robust feature extraction and classification tool.

## Results and discussion

Table 11 depicts the experimental setup for the proposed work, followed by the assessments using standard assessment metrics and Python with TensorFlow framework support to implement the proposed model.

**Table 11:** System configuration details used in this proposed work.

Types	Specification
Processor	12th Gen Intel(R) Core (TM) I9-12900 K 3.20 GHz
RAM	32.0 GB (31.7 GB usable)
System Type	64-bit operating system, x64-based processor

The segmentation efficiency is assessed using Dice Coefficients for the input patched images. Let A be the set of segmented pixels in the mask, and B be the set of pixels in the ground truth mask. The notation |A ∩ B| represents the number of overlapping pixels, while |A| and |B| indicate the total number of pixels in the segmented and ground truth masks, respectively.

9 $$\:\text{D}\text{i}\text{c}\text{e}\:\text{C}\text{o}\text{e}\text{f}\text{f}\text{i}\text{c}\text{i}\text{e}\text{n}\text{t}\:=\:\frac{2*|A\cap\:B|}{\left|A\right|+\left|B\right|}$$

The experimental model was assessed for accuracy, recall, F1 score, and precision. Precision and recall metrics are derived from true positives (TP), false positives (FP), and false negatives (FN).

10 $$\:\text{P}\text{r}\text{e}\text{c}\text{i}\text{s}\text{i}\text{o}\text{n}\:=\:\frac{TP}{\:\:\:\text{T}\text{P}+\text{F}\text{P}}$$

11 $$\:\text{R}\text{e}\text{c}\text{a}\text{l}\text{l}\:=\:\frac{TP}{\:\:\:\text{T}\text{P}+\text{F}\text{N}}$$

The F1-score metric is calculated based on precision and recall.

12 $$\:\text{F}1-\text{s}\text{c}\text{o}\text{r}\text{e}\:=2\text{*}\frac{Precision*Recall}{\text{R}\text{e}\text{c}\text{a}\text{l}\text{l}+\text{P}\text{r}\text{e}\text{c}\text{i}\text{s}\text{i}\text{o}\text{n}}$$

The measured precision counts pathological annotations, and the model’s test outcomes are appropriately validated.

13 $$\:\text{A}\text{c}\text{c}\text{u}\text{r}\text{a}\text{c}\text{y}\:=\:\frac{TP+TN}{\text{T}\text{P}+\text{T}\text{N}+\text{F}\text{P}+\text{F}\text{N}}$$

Specificity measures the effectiveness of test results correctly, categorizing the true positive (TP)

14 $$\:\text{S}\text{p}\text{e}\text{c}\text{i}\text{f}\text{i}\text{c}\text{i}\text{t}\text{y}\:=\:\frac{TP}{\:\:\:\text{F}\text{P}+\text{F}\text{N}}$$

15 $$\:\text{A}\text{U}\text{C}\:=\:\frac{1}{\:\:2}\left(\frac{TP}{\:\:\:\text{T}\text{P}+\text{F}\text{N}}\:+\:\frac{TN}{\:\:\:\text{T}\text{N}+\text{F}\text{P}}\right)$$

The Area Under the Curve (AUC) metric is a comprehensive evaluation tool for assessing a model’s performance. It analyses how effectively the model distinguishes between different classes by examining various methods used in classification tasks. By calculating the area under the Receiver Operating Characteristic (ROC) curve, the AUC provides insight into the model’s ability to correctly classify positive and negative instances across a range of thresholds, ultimately ensuring that the resulting classification outputs are reliable and informative.

### Deep U-Net SAM model

**Fig. 12:**
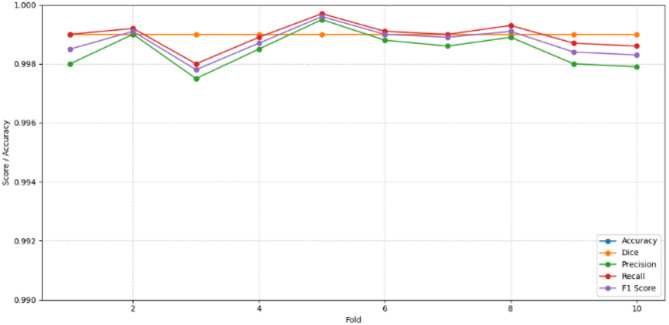
Performance metrics graph for the proposed Deep U-Net SAM model evaluated 10-fold cross-validation.

The CCP process for nuclei detection utilizes the Deep U-Net SAM model, which accurately locates and identifies nuclei and calculates their density using microns per pixel measure in malignant patches. The model is trained for 50 epochs and tested using both ground truth and predicted masks, with an accuracy of 99.08% using 10-fold cross-validation. From the 5th fold, the model got improved in high accuracy as shown in Fig. 12, categorizing the nuclei into low, medium, and high-density groups.

Table 12 presents a comparative analysis of the segmented results images achieved by the proposed Deep U-Net SAM model alongside existing models. This table highlights the distinctive features and performance of each model in detail. Furthermore, Table 13 offers a quantitative assessment of the Deep U-Net SAM model, illustrating its effectiveness compared to other segmentation models and alternative attention-based approaches utilized in medical image analysis. This comprehensive evaluation underscores the advantages and improvements brought forth by the proposed model in this critical field.

**Table 12:**
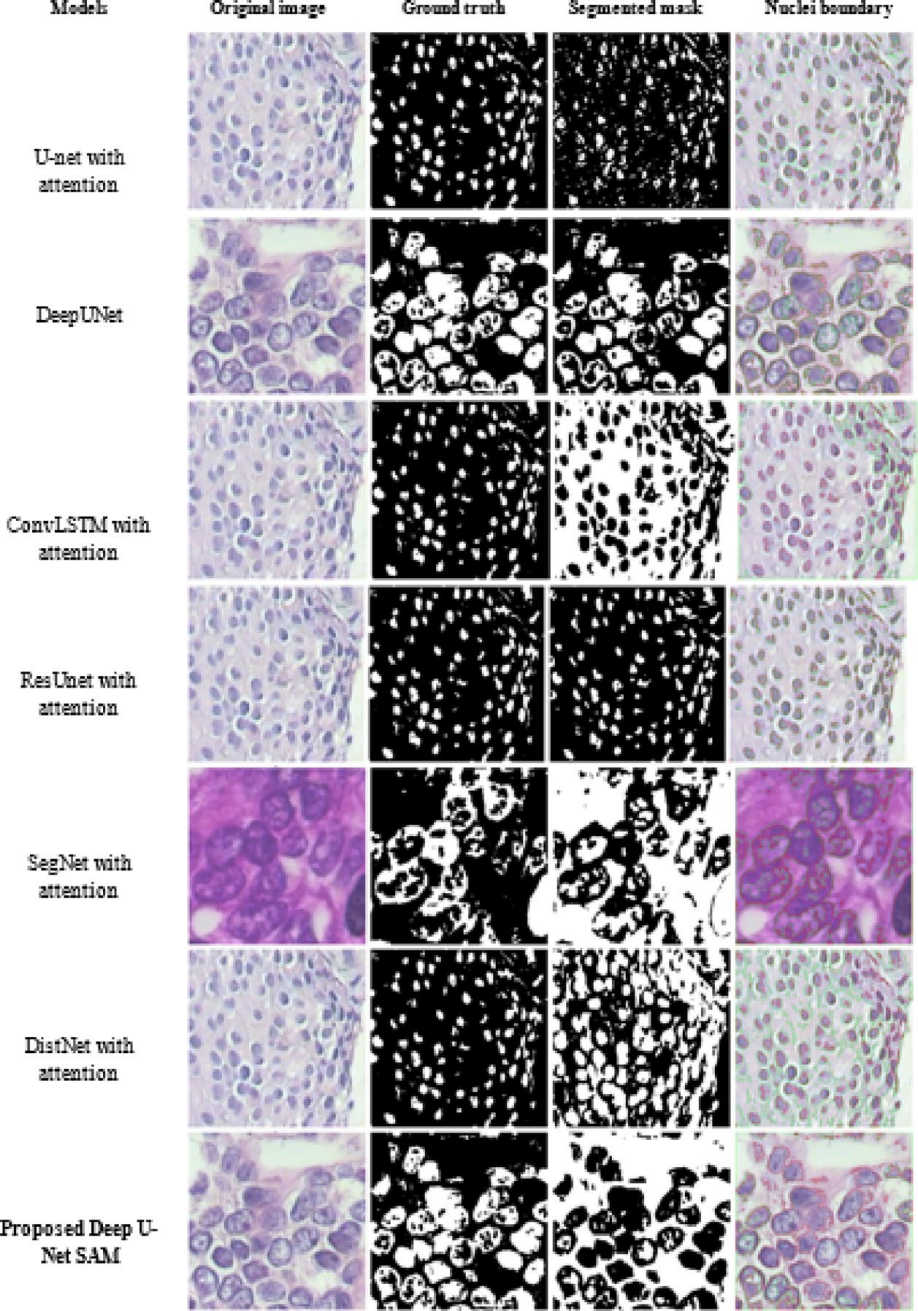
Illustrates a comparison of segmented results images for the proposed deep U-Net SAM and existing models.

**Table 13:** Quantitative assessment of the proposed deep U-Net SAM model with the existing segmentation model.

Models	Dataset	Images	F1-score	Accuracy	Precision	Recall	Dice coefficient		Computational time per image in seconds)
U-net with attention	BreakHis	Histopathology (BC)	94.34%	91.73%	99.75%	99.79%	98.45%		1.6s
DeepUnet	BreakHis	Histopathology (BC)	88.33%	81.83%	95.02%	94.32%	97.94%		2.0s
ConvLSTM with attention	BreakHis	Histopathology (BC)	93.14%	94.10%	91.63%	90.02%	90.16%		1.9s
ResUnet with attention	BreakHis	Histopathology (BC)	94.15%	92.98%	93.02%	90.12%	96.66%		1.8s
SegNet with attention	BreakHis	Histopathology (BC)	95.09%	96.09%	89.04%	90.02%	96.71%		1.7s
DistNet with attention	BreakHis	Histopathology (BC)	95.59%	85.32%	89.02%	88.02%	97.77%		–
ACX U-Net^60^	LUNA-16	Lung cancer(CT)	–	0.98	0.97	0.98	–		–
3D U-Net^61^	LUNA-16	Lung cancer(CT)	–	–	0.74	–	0.84		–
IOMT-assisted modified U-Net^62^	LIDC-IDRI	Lung cancer (CT)	0.86	–	0.74	0.86	0.85		–
DS-Trans U-Net^63^	LIDC-IDRI	Lung cancer (CT)	0.92		0.90	0.94	0.86		–
Proposed deep U-Net SAM			99.04%	99.04%	99.75	99.89%	99.90%		1.4s

### SNN models

The CCP process intricately examines malignant patches, analyzing their characteristics to classify them into distinct groups based on low, medium, and high nuclei density. This categorization helps to enhance our understanding of the tumor’s behaviour and potential aggressiveness. The model is then trained and tested on these categorized patches. In contrast, benign overlay patches are fed directly into the model. However, the model’s ability to learn from malignant patches is limited, which affects its overall training effectiveness. Our goal is to demonstrate that magnifications of 40x and 400x do not impact classification more than the category itself. Therefore, SCNN and DCNN were trained and tested for the lowest and highest magnification settings.

The proposed models, SCNN and DCNN, utilize the BreakHis dataset, which contains histopathology images at 400x and 40x magnifications. The dataset is divided into 224*224 pixel overlapping patches for benign images, while patches for malignant nuclei are based on micron-per-pixel measurements. The dataset is split into training (70%), validation (15%), and testing (15%) sets, featuring various magnification levels and density classifications: low, medium, and high. Table 14 outlines the parameters used in the SNN models. The learning rate starts at 0.01 and increases with a decay rate of 0.8. While accuracy improved with weight updates up to 2*$\:{10}^{-5}$training progress declined after ten epochs, requiring backpropagation corrections. The learning rate was inadequate for varying density patches, leading to the recommendation of a DCNN model.

**Table 14:** SNN model parameter settings used during the training process.

Parameter	Configuration	
	SCNN	DCNN
Activation function	Softmax	Softmax
Learning rate	1e-5 to 1e-8 maximum	1e-4 to 1e-8 maximum
Epochs	50	50
Batch size	32	32
Optimizer	Adam	Adam
Loss function	categorical cross-entropy	categorical cross-entropy
Early stopping	Delta values-0.01, patience-5, Verbose-1, mode-auto, decay-0.8	Delta values-0.001, patience-10, Verbose-1, mode-auto, decay-0.8

In the DCNN, the learning rate scheduler adjusts the initial rate from 0.001 to a maximum of 1, decaying to a minimum of 0.8. The DCNN performed better than the SCNN by updating weights up to 4. It showed improved testing and classification performance across different magnifications (400× and 40×), as detailed in Tables 15 and 16, proving more effective in classifying malignant patches in breast cancer density images.

**Table 15:** Performance metrics of the proposed methodologies for classifying benign and malignant classes using test sets with various densities.

Metrics	400×						40×					
	SCNN			DCNN			SCNN			DCNN		
	LD	MD	HD	LD	MD	HD	LD	MD	HD	LD	MD	HD
Precision	0.91	0.77	0.75	0.93	0.92	0.83	0.89	0.87	0.84	0.99	0.99	0.97
Recall	0.91	0.77	0.75	0.93	0.92	0.82	0.88	0.87	0.83	0.99	0.99	0.97
1-score	0.91	0.77	0.75	0.93	0.92	0.82	0.88	0.87	0.83	0.99	0.99	0.97
AUC	0.96	0.86	0.85	0.98	0.97	0.91	0.95	0.94	0.92	1.00	1.00	1.00

**Table 16:** Performance metrics of the proposed methodologies include overall accuracy, training, validation, and testing performance.

Metrics	400× (accuracy)						40× (accuracy)					
	SCNN			DCNN			SCNN			DCNN		
	Train	Validation	Test	Train	Validation	Test	Train	Validation	Test	Train	Validation	Test
Low	0.80	0.82	0.82	0.92	0.93	0.93	0.89	0.90	0.91	0.96	0.98	0.99
Medium	0.74	0.75	0.77	0.90	0.91	0.92	0.84	0.86	0.87	0.96	0.98	0.99
High	0.74	0.75	0.75	0.80	0.81	0.82	0.80	0.82	0.83	0.94	0.96	0.97

**Fig. 13:**
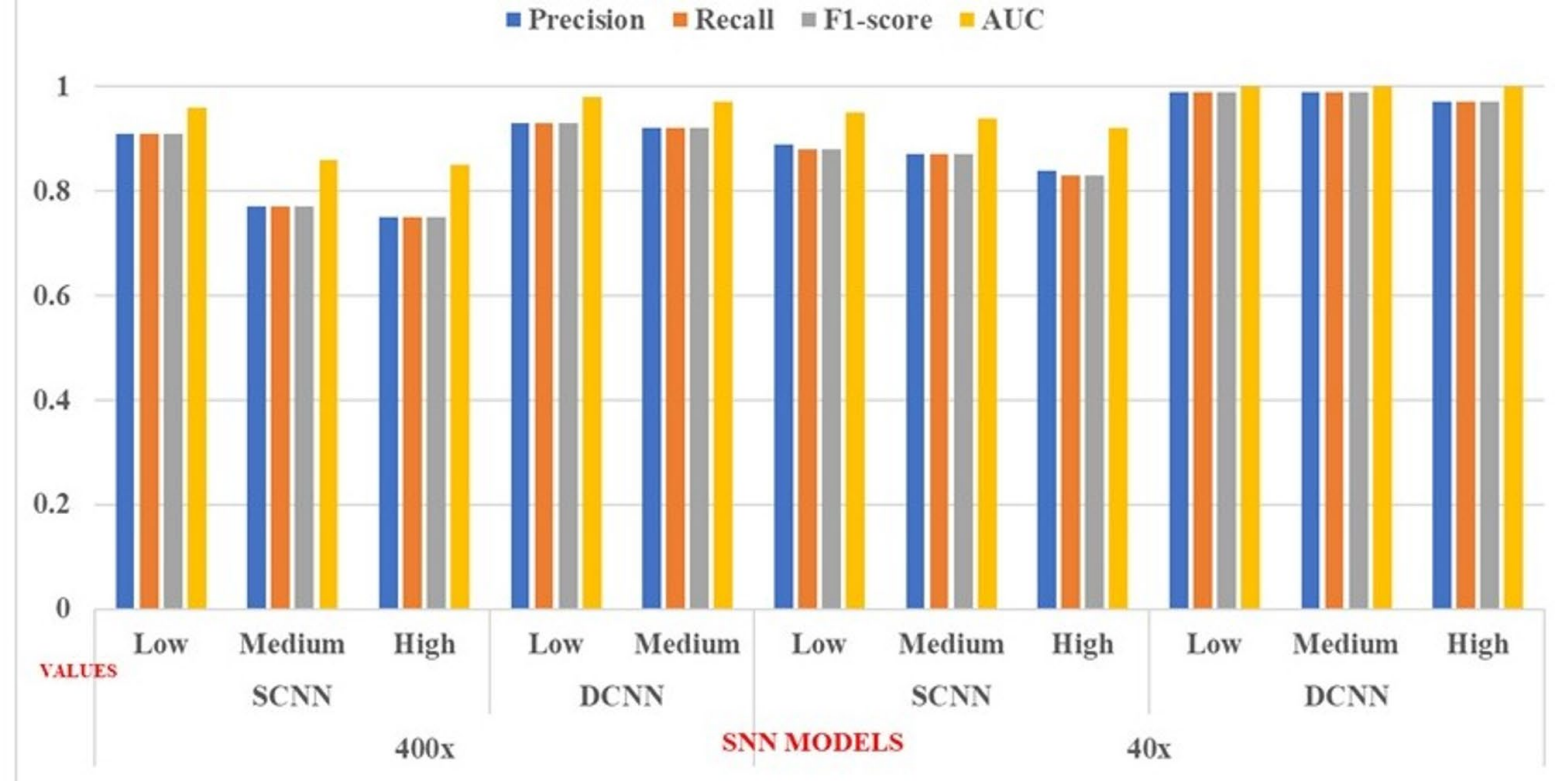
Performance metrics of the proposed model SNN.

**Fig. 14:**
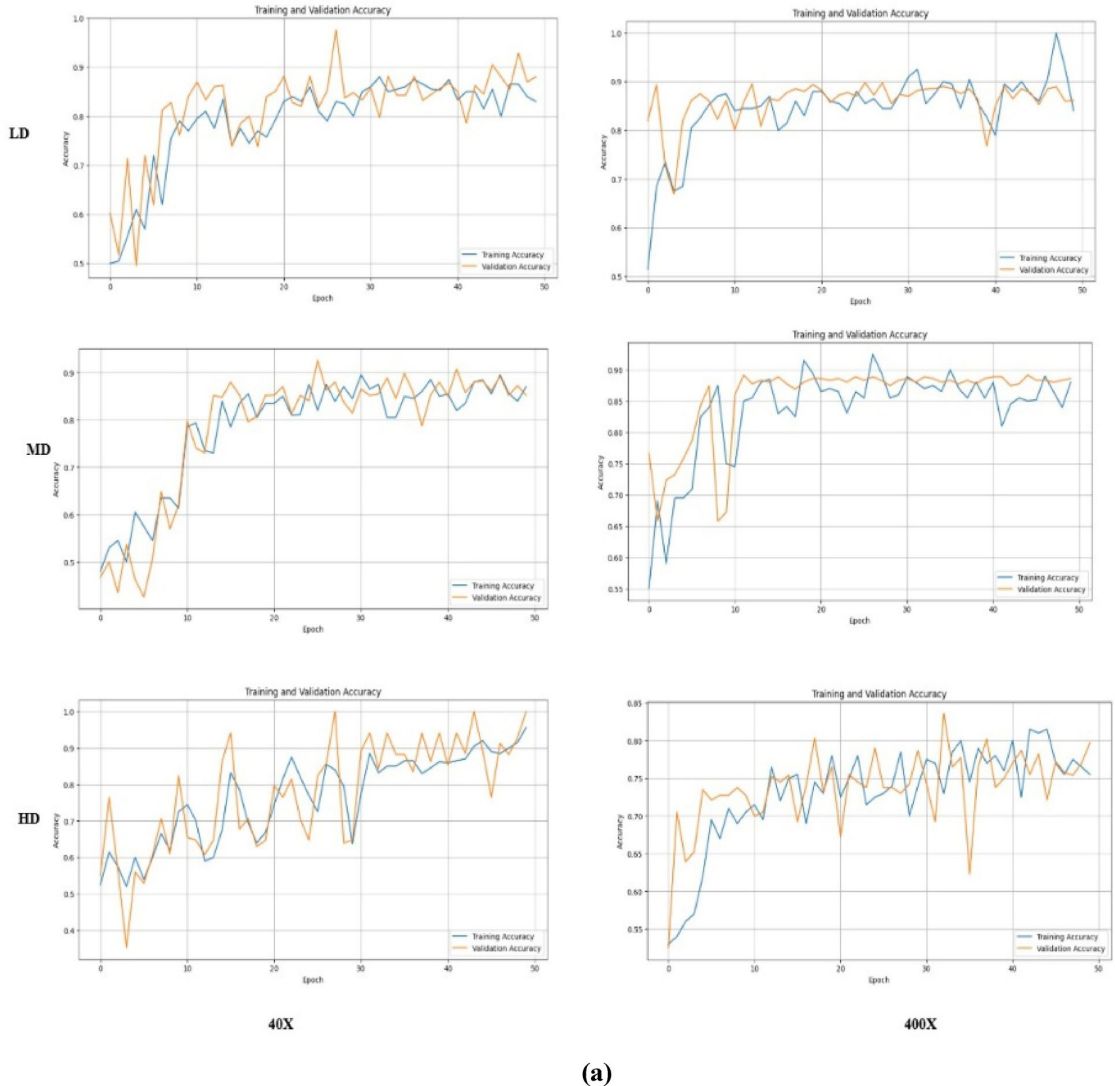

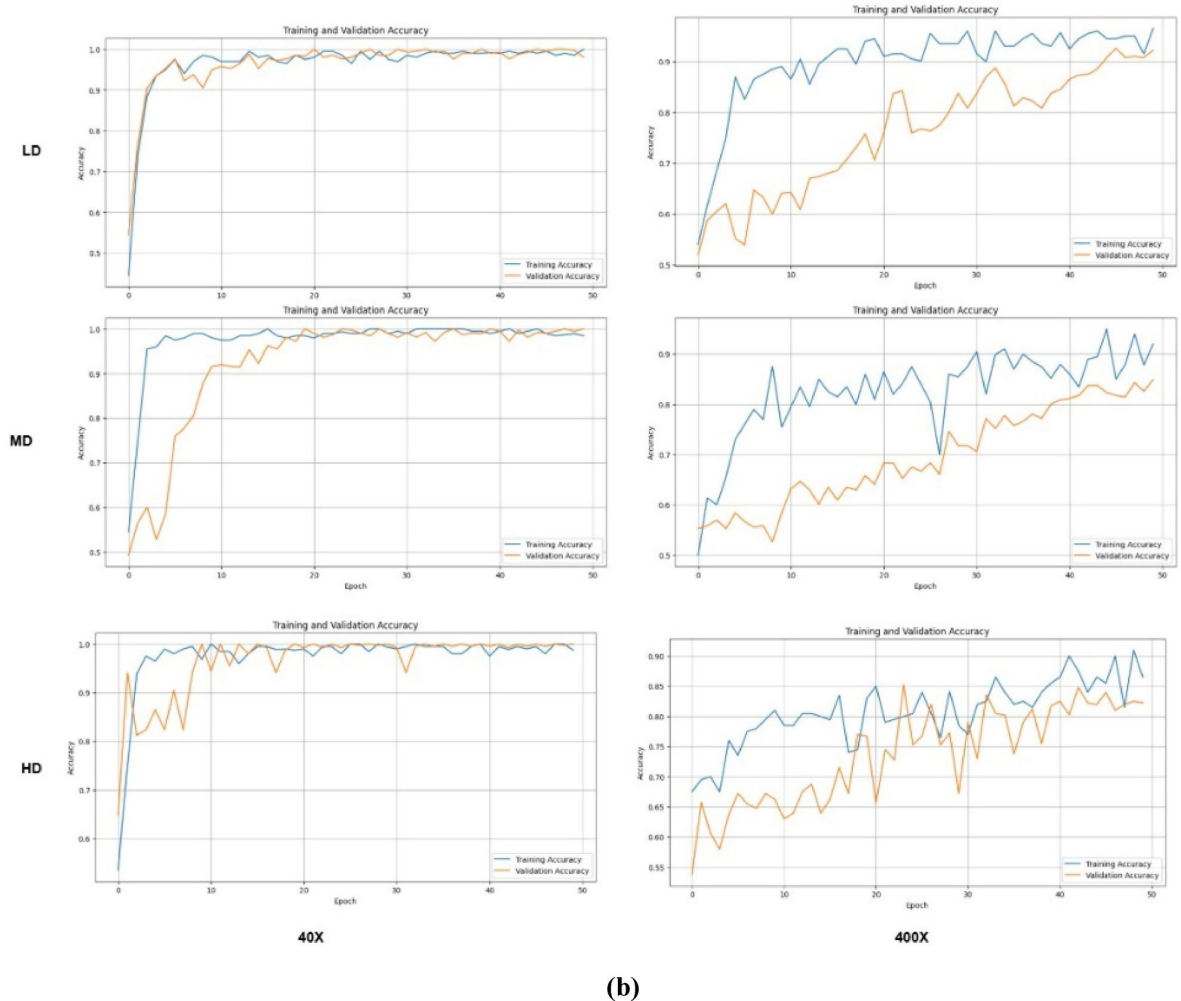
Training and validation results of SNN models (**a**) SCNN and (**b**) DCNN.

Figure 13 illustrates the F1 score graph for both the SCNN and DCNN models, indicating that the DCNN model has significantly enhanced its classification capabilities. Meanwhile, Fig. 14 depicts the training and validation accuracy of the proposed model. The DCNN successfully classifies benign and malignant image patches of varying densities without misclassification. The overall classification accuracy for nuclei-density patched images at different magnifications is as follows: 0.98 for low density, 0.97 for medium density, and 0.91 for high density, with perfect scores of 1.0 in other categories.

Table 17 presents the numerical values for true positives (TP) and false positives (FP) associated with various densities of the proposed SNN models at different magnifications (40× and 400×). Tables 18 and 19 visually display their confusion matrices and AUC graphs, while Table 20 visually represents the precision and recall graphs of 400x magnification. The results show that the SCNN model recorded false negative and false positive rates of 0.04 and 0.05 at magnifications of 400× and 40×, respectively, for low-density nuclei. In comparison, the DCNN achieved false negative and false positive rates of 0.02 and 0.01. The SCNN model noted false negative and false positive rates of 0.0 and 0.08 for high-density patches, while the DCNN indicated rates of 0.09 and 0.0.

**Table 17:** True positive (TP) and false negative (FN) results for 40× and 400× magnification.

Model	Density	TN	TP	FP	FN	Benign	Malignant	Total samples
SCNN (40×)	LD	398	342	22	77	420	419	839
	MD	245	195	31	34	276	229	505
	HD	184	162	48	21	232	183	415
SCNN (400×)	LD	304	281	19	39	323	320	643
	MD	136	132	39	41	175	173	348
	HD	343	299	88	129	431	428	859
DCNN (40×)	LD	416	415	4	4	420	419	839
	MD	270	229	6	0	276	229	505
	HD	218	183	14	0	232	183	415
DCNN (400×)	LD	306	291	17	29	323	320	643
	MD	167	152	8	21	175	173	348
	HD	331	376	100	52	431	428	859

**Table 18:**
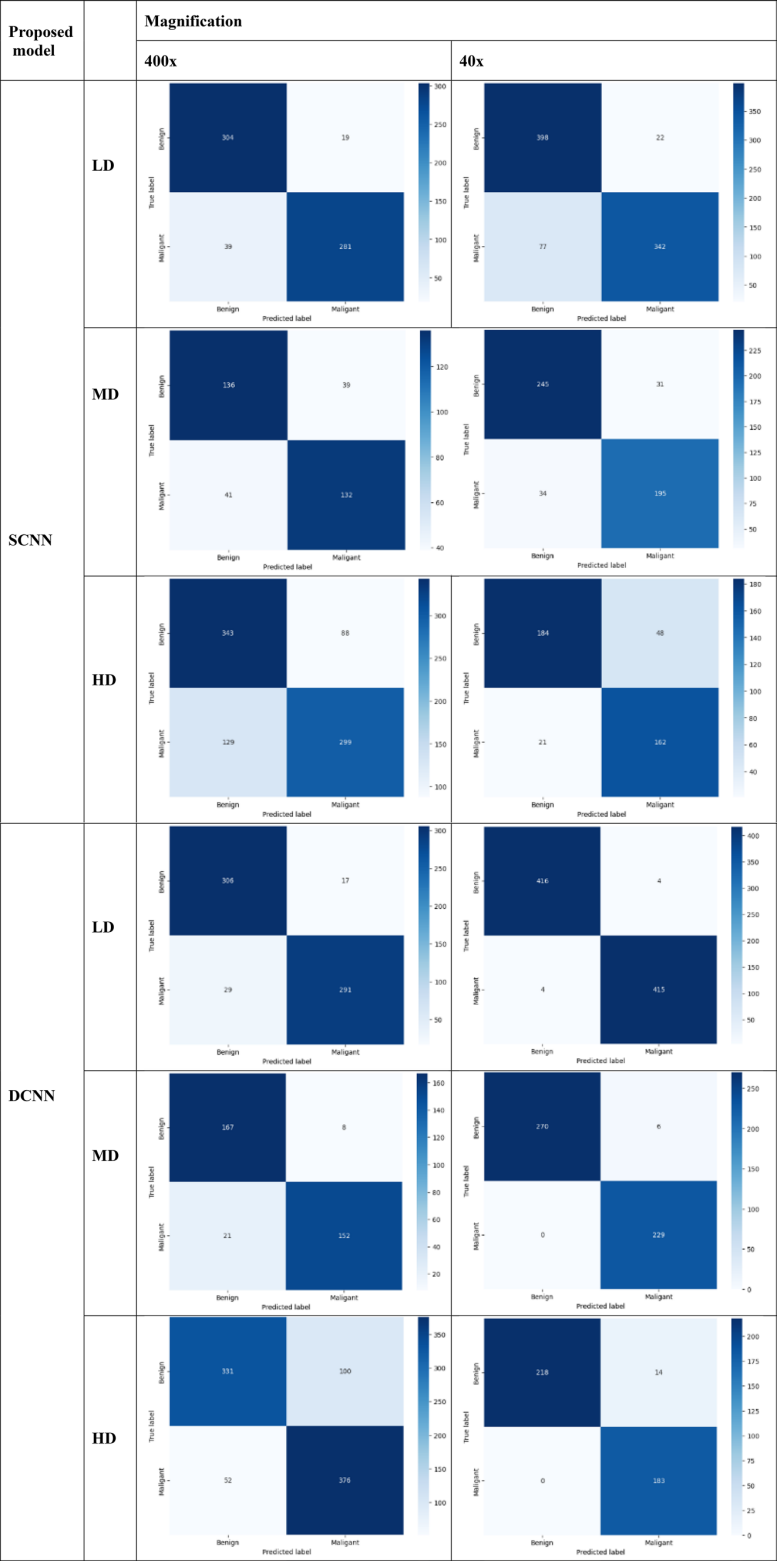
Confusion matrix of proposed models at different magnifications.

**Table 19:**
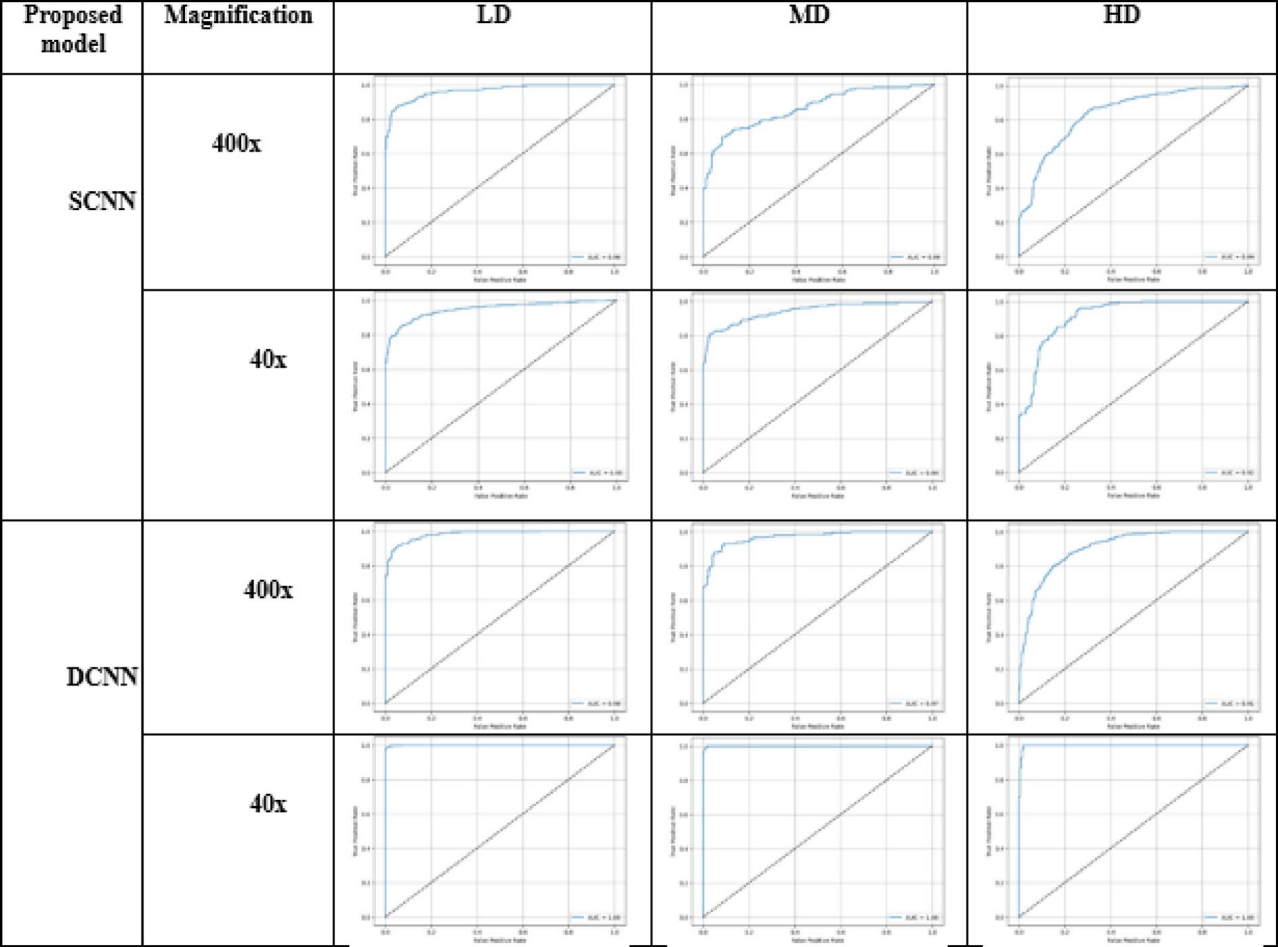
AUC graph of proposed models at different magnification levels.

**Table 20:**
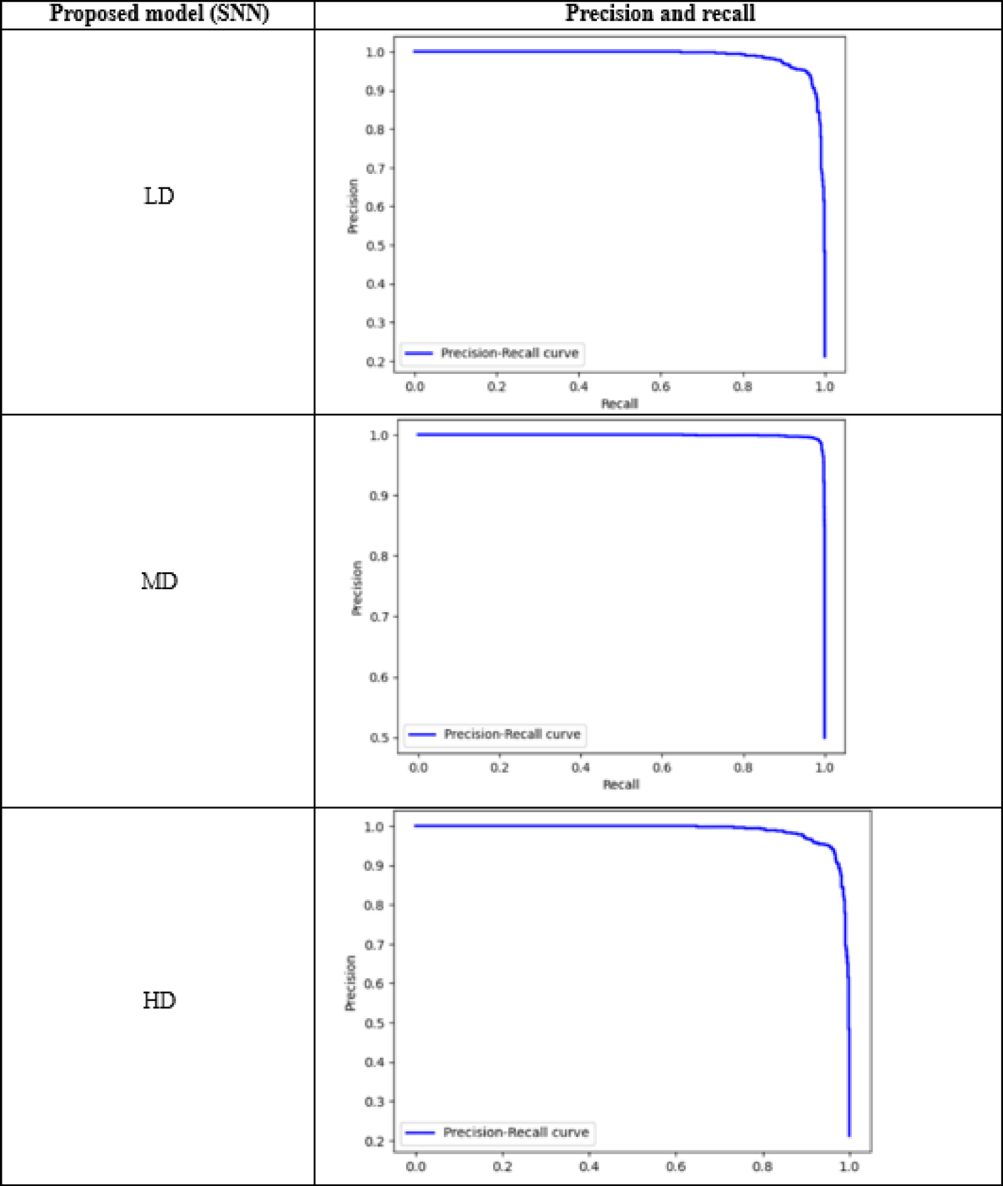
Precision and recall graph of the proposed SNN models of 400× magnification.

## Discussion

To evaluate the performance of the Deep U-Net SAM model for nuclei segmentation and density calculation in malignant images, we compared it with several existing models that utilize attention mechanisms, as mentioned before in Table 13. The models we examined include U-Net with attention, DeepUNet, Conv-LSTM with attention, ResUnet with attention, DistNet with attention, and SegNet with attention. These models employ hierarchical attention mechanisms and achieve F1-scores ranging from 88 to 95%, accuracies between 81% and 96.04%, precision rates from 89 to 99%, recall rates spanning from 88 to 99.79%, and Dice coefficients varying from 90 to 98%. In contrast, our proposed model achieved exceptional results with an F1-score of 99.04%, accuracy of 99.04%, precision of 99.75%, recall of 99.89%, and a Dice coefficient of 99.90%. Additionally, our model, when compared to the existing models and trained on other medical image datasets such as CT images of lung cancer from the LUNA and LIDCR datasets, demonstrated superior performance in F1-score, precision, recall, and Dice coefficient.

**Table 21:** Comparison of dice coefficient accuracy across different magnifications levels with existing models.

Models	Year	Dice coefficient
Dilation, Residual and Dense block-U-Net (DRD)^35^	2024	93%
Transformer based Spatial-Channel Attention (TSCA-Net)^36^	2024	82%
Densely convolutional Breast U-shaped Network (BU-Net)^45^	2024	88.9%
U-Net with Annotator network^46^	2024	78%
Multi-Attention Transformer Network (MATNet)^47^	2024	91.90%
Multi Stage Nuclie Segmentation (MSNSeg)^48^	2024	79%
Multi-scale Adaptive Convolutional Network with Dual Decoders (MSAC-DD)^49^	2025	98%
Proposed Deep U-Net SAM model	-	99.90%

As shown in Table 21, the proposed model, Deep U-Net SAM, shows enhanced efficiency in identifying nuclei boundaries compared to the existing model, obtaining a Dice coefficient with a ground truth accuracy of 99.90%. Dilation, Residual and Dense block-U-Net (DRD)^35^ network, characterized by the incorporation of depthwise in the residual block and spatial pooling, has demonstrated exceptional segmentation performance. The network exhibited a Dice coefficient of 93%, Transformer-based Spatial-Channel Attention (TSCA-Net)^36^ yields an impressive Dice coefficient of 82%. This model excels in the precise detection and classification of the nuclear region. However, the computational cost is relatively high compared with that of other models. U-Net with Annotator network^46^ is an advanced segmentation model that attains a Dice coefficient of 78%, and Multi-Attention Transformer Network (MATNet)^47^, the model accurately distinguishes nuclear and non-nuclear regions, achieving a Dice coefficient of 91.90% when it is applied to other medical imaging datasets. The incorporation of pixel shuffling enhances segmentation by integrating multiscale feature fusion and extracting significant feature channels from the input. Multi-scale Adaptive convolutional Network with Dual Decoders (MSAC-DD)^49^ and Multi-scale Adaptive convolutional Network with Dual Decoders (MSAC-DD)^49^ exhibit the dice coefficient of 79–98% compared to proposed model.

**Table 22:** Comparison of performance metrics between the proposed SNN and the existing models.

Classifier	Accuracy	Precision	Recall	F1-score
RESNet50	75%	70%	78%	67%
VGG16	77%	72	77	70
VGG19	89%	89%	90%	86%
DenseNet201	80%	75%	75%	72%
DenseNet169	78%	74%	79%	69%
Proposed SCNN	99.03%	99%	99%	99%
Proposed DCNN	99.92%	99.80%	99.76%	99.90%

To improve the learning process of the BreakHis image dataset, patches were trained and evaluated using a pre-existing model without the CCP method. The pre-existing model had difficulty identifying boundaries of overlapping nuclei, limiting its capacity to learn more effectively. Consequently, the accuracy varied from 77 to 89%, in contrast to other existing models, as illustrated in Table 22.

Table 23 details the performance of existing models and highlights the ease of learning from the input images. The EfficientNet2 Self-Supervised Constructive learning method^50^ struggled to learn effectively from 40x magnification images and exhibited issues with computational time, achieving an accuracy of 93.63%. With depth-wise separable convolution and a swim transformer^51^ in the encoder block, the model improved its accuracy to 98.13% while surpassing more boundary regions of the overlapped nucleus, resulting in precision, recall, and F1-scores of 98%, 97%, and 98%, respectively. The Spatial Pyramidal Complex Zernike (SPCZP) moments-based pooling method^56^ utilized a pooling module combined with channel and spatial attention mechanisms in the decoder, showcasing outstanding exponential performance with an accuracy of 94.16% and precision of 96.38%. Compared to all pretrained and fusion models, our proposed model learned more efficiently by implementing the intelligent learning process of CCP inputs with high, low, and medium density nuclei patches, demonstrating improved efficiency in overlapping nuclei patches of high density, though it faced some challenges with boundary detection of nuclei, ultimately achieving an overall accuracy of 99.92%.

**Table 23:** Comparison of classification accuracy between the proposed method and existing methods.

Model	Year	Accuracy	Precision	Recall	F1-score
EfficientNet2 Self-supervised Constructive learning method^50^	2024	93.63%	–	–	–
Depth-wise separable convolution and swim transformer^51^	2024	98.13%	98%	97%	98%
Levenberg-Marquardt based Deep Neural Network (LMHisNet)^52^	2024	99%	99%	99%	99%
Deep Belief Network Based CNN(DBN)^53^	2024	98%	97%	97%	97%
Breast Cancer Histopathology Images Convolutional Network (BCHI-ConvNet)^54^	2024	99.75%	98.75%	98.79%	98.70%
Self-Learning Deep Neural Network (DNN)^55^	2024	99.1%	–	–	–
Spatial Pyramidal Complex Zernike (SPCZP) moments-based pooling approach^56^	2024	94.16%	–	–	96.38%
Ensemble ResNet50^57^	2024	98%	–	–	–
Multi-scale Dual Adaptive Attention (MDAA)^58^	2024	96%	–	–	–
Fully Convolutional Spatial Channel with Attention mechanism (FCSCAM)^59^	2024	91.25%	–	–	–
Proposed SCNN	-	99.03%	99%	99%	99%
Proposed DCNN		99.92%	99.80%	99.76%	99.90%

### Ablation study

**Fig. 15:**
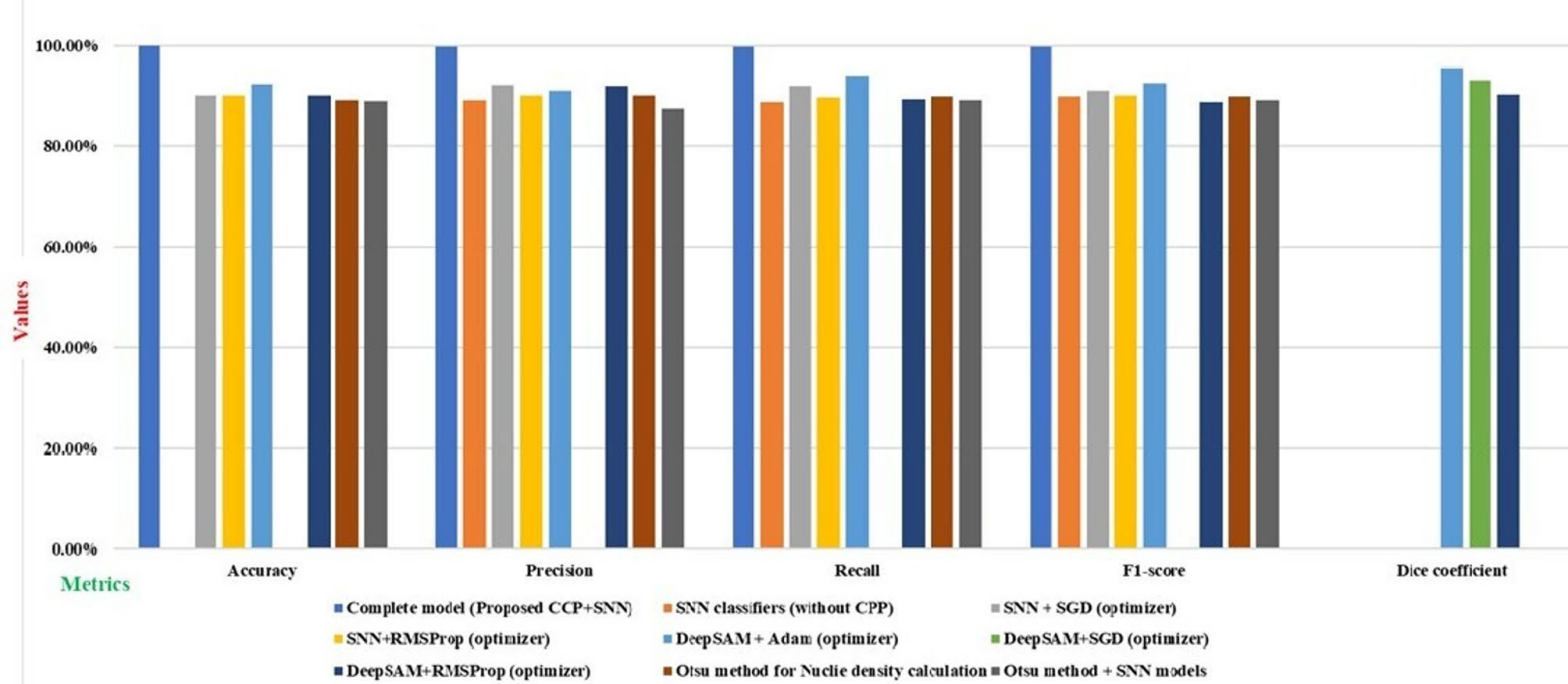
Performance metrics of the proposed CCP + SNN models-based ablation components.

Table 24; Fig. 15 display the results from an ablation study performed on the BreakHis dataset, which contains histopathology images associated with breast cancer. The findings indicate that integrating different techniques applied to the input images has a substantial effect.

**Table 24:** Ablation study of the proposed model and its associate challenges.

Model variations	Accuracy	Precision	Recall	F1-score	Dice coefficient	Comments
Complete model (Proposed CCP + SNN) with Adam optimizer	99.92%	99.80%	99.76%	99.90%	**-**	The hybrid model extracted malignant nuclei features, such as boundary size, and calculated the density, and effectively classified the samples without misclassification of benign samples.
SNN classifiers (without CPP)	90.20%	89.20%	88.90%	89.99%	**-**	Without (CPP), the model fails to deeply understand the data’s features, leading to misclassifications between benign and malignant cases. This hampers its ability to differentiate accurately, impacting prediction reliability.
SNN + SGD (optimizer)	90.14%	92.20%	92.15%	91.20%	**-**	The SNN classifier effectively extracts relevant features from the data but struggles with accurately categorizing certain samples, leading to many misclassifications.
SNN + RMSProp (optimizer)	90.20%	90.15%	89.90%	90.14%	**-**	The SNN classifier extracts features but struggles with misclassifying benign and malignant samples.
DeepSAM + Adam (optimizer)	92.50%	91.20%	94.20%	92.60%	95.75%	The SAM model effectively identified more nuclei and calculated their density accurately. These nuclei show remarkable efficiency.
DeepSAM + SGD (optimizer)	**-**	**-**	**-**	**-**	93.21%	Lack of nuclei boundary
DeepSAM + RMSProp (optimizer)	90.20%	92.02%	89.50%	88.87%	90.50%	More significant nuclei and smaller nuclei are not detected.
Otsu method for Nuclie density calculation	89.20%	90.20%	90%	90.%	-	The more prominent nuclei are clearly observed, while the smaller nuclei at the boundary remain undetected, leading to a noticeable deficiency in their overall count.
Otsu method + SNN models	89%	87.60%	89.20%	89.20%	-	The Otsu method failed to identify a greater number of nuclei within the patches of images categorized as low, medium, and high density. As a result, the model was unable to classify the samples effectively.

This comprehensive strategy improves the models’ capacity to learn more efficiently, ultimately enhancing their effectiveness in accurately analyzing and interpreting histopathology images related to breast cancer. The proposed complete model (Proposed CCP + SNN) using the Adam optimizer achieves a maximum accuracy of 99.92%. The hybrid model successfully extracted features of malignant nuclei, such as boundary size, calculated their density, and accurately classified samples without misclassifying benign cases. In contrast, the SNN classifiers (when CPP is not included) struggle as the model lacks a deep understanding of the data’s features, leading to misclassifications between benign and malignant cases. This limitation adversely affects its capacity to differentiate accurately, compromising the reliability of the predictions.

The SNN combined with SGD and SNN with RMSProp optimizers achieved accuracies of 90.14% and 90.20%, respectively, but still exhibited shortcomings. While the SNN classifier effectively extracts essential features from the data, it faces challenges in accurately categorizing some samples, resulting in a significant number of misclassifications. The SNN classifier captures features but encounters difficulties in distinguishing benign and malignant samples accurately. The DeepSAM model with the Adam optimizer recorded a dice coefficient of 95.75%, demonstrating its ability to clearly identify the nuclei and categorize their densities as low, medium, or high for the training model classification.

The SAM model successfully recognized a more significant number of nuclei and efficiently calculated their density. With DeepSAM combined with the SGD and RMSProp optimizers achieving dice coefficients of 93.21% and 90.50%, respectively, there was still a failure to detect nuclei boundaries, with some larger and smaller nuclei being overlooked. The Otsu method (without utilizing the DeepSAM model) in CCP for nuclei detection revealed prominent nuclei distinctly, but smaller nuclei at the boundary went undetected, resulting in a noticeable deficiency in their aggregate count, achieving an accuracy of 89.20%. The Otsu method combined with SNN models (excluding DeepSAM) underperformed in recognizing a larger number of nuclei within the patches of images categorized as low, medium, and high density, ultimately failing to classify the samples effectively, with an accuracy of 89%. This ablation study provides remarkable contributions from the proposed hybrid model of CCP and SNN, which enhances the learning process intelligently by utilizing efficient inputs of CCP and classifies breast cancer histopathology images from the BreakHis dataset without misclassification of benign and malignant samples, making it valuable for clinical diagnostic tools.

### Statistical analysis

**Fig. 16:**
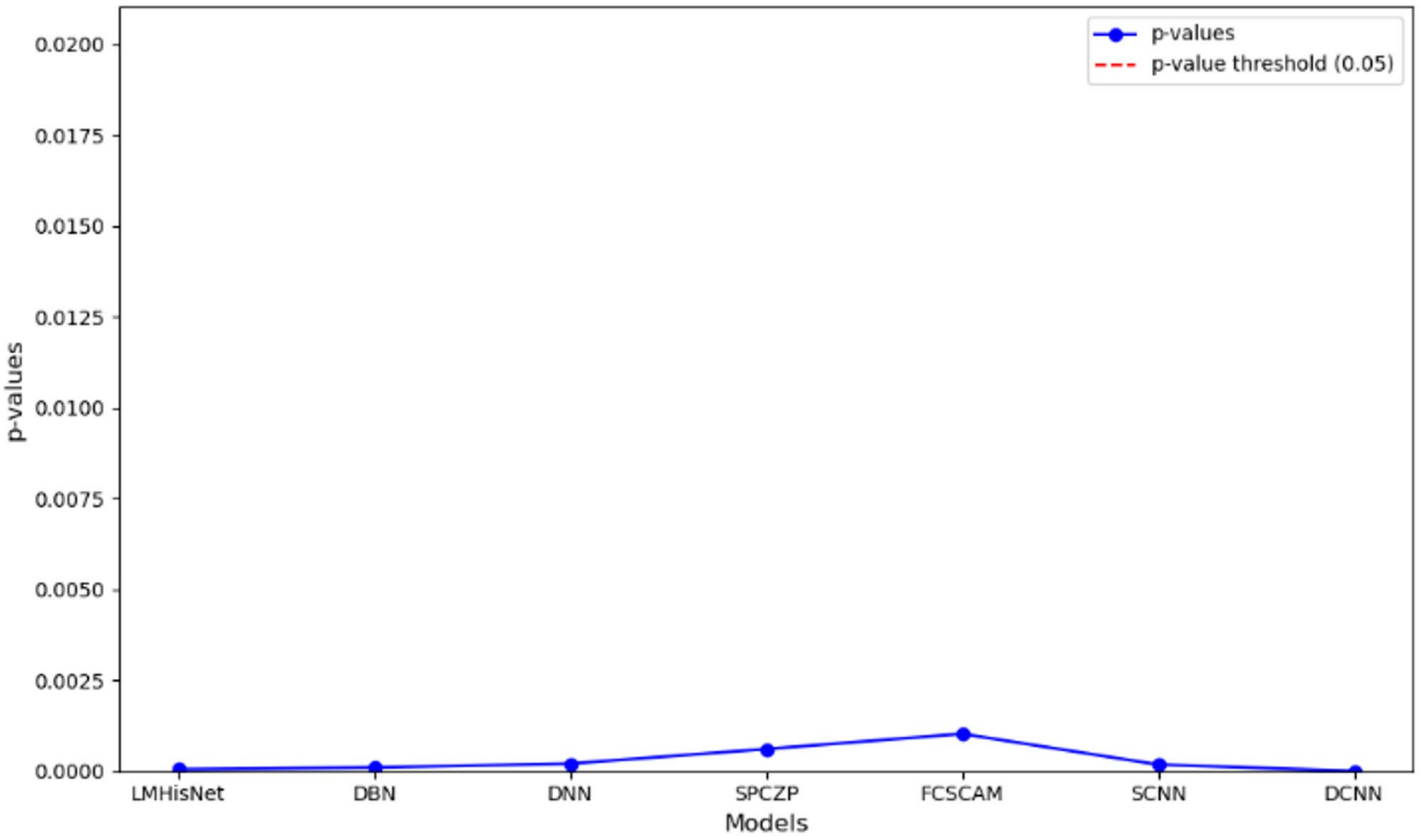
Comparison of p-values for the proposed model SNN versus other models.

**Fig. 17:**
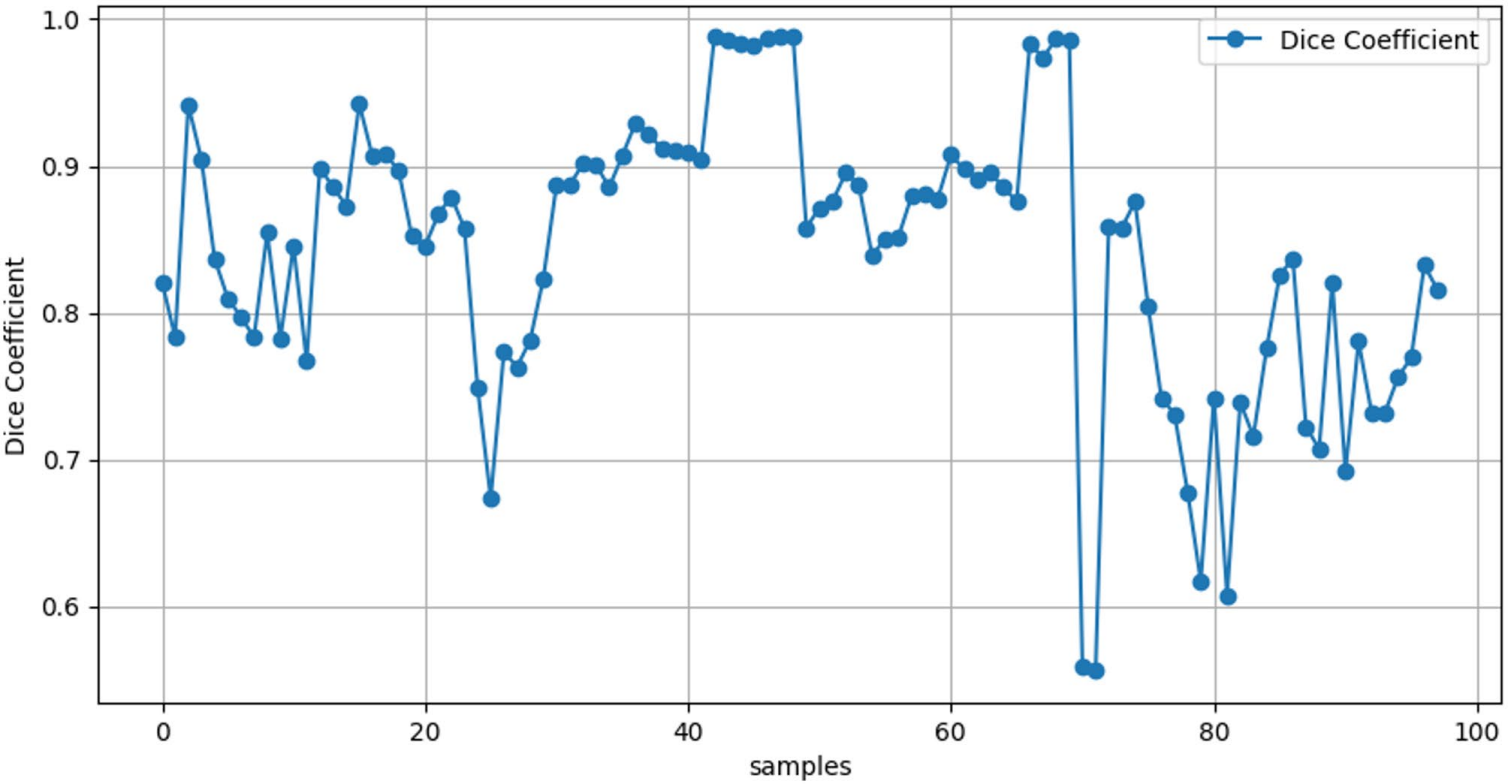
Confidence intervals of the BreakHis datasets based on the Dice coefficient.

Statistical analysis is performed for the proposed model SNN via p-values, and confidence intervals are assessed for hundreds of samples of Deep U-Net SAM model to prove the reliability and variability of the model. The confidence intervals of the BreakHis datasets are assessed via the efficient metric dice-coefficient for hundreds of samples, and their performance graphs are shown in Figs. 16 and 17, and the numeric values are represented in Table 25.

**Table 25:** *P*-values from a paired *t*-test comparing our models with other methods.

Metrics	LMHisNet	DBN	DNN	SPCZP	FCSCAM	SCNN	DCNN
*P*-value	0.00005	0.0002	0.00019	0.0006	0.001	0.00018	0.000039

## Conclusion

The proposed work highlights the importance of preprocessing in influencing the learning process of classification models. To accomplish this, the CCP and SNN models work together using BreakHis datasets of different magnifications (40× and 400×). The CCP facilitates the SNN models by refining the essential features necessary for classification. Micron per pixel measurement efficiently localizes the nuclei in each patch and calculates the nuclei density. A defined threshold helps to categorize the patches as low, medium, and high. The proposed work addresses the challenges in histopathology images through CCP and SNN. The motive of the proposed work is to make the model learn the features effectively and classify accurately for easy cancer detection. State-of-the-art detects and segments the nuclei efficiently with a 99.90% dice coefficient and a classification accuracy of 99.03–99.92% at different magnifications. The proposed method can support the real-time slide images to diagnose the cancer with fewer false positives and true negatives and to reduce the pathologist’s time complexity.

### Limitations of the proposed method

The dataset poses challenges for achieving objective outcomes due to image clarity issues, which can lead to ambiguous details and complicate analysis. A defined threshold categorizes patches as low, medium, and high, but this is a limitation for CCP techniques. The variation in micron measurements for nuclei localization depends on image magnification (40× vs. 400×) and is influenced by scanner hardware, impacting image quality. At 40× magnification, a micron measurement of 0.1625 provides less detail, making it harder to identify smaller nuclei, which can affect accurate diagnosis. Our Deep U-Net SAM segmentation model struggles to separate overlapping nuclei, and its performance at 40× is significantly less effective than at 400×. Additionally, our SCNN model faces difficulties analyzing high-density nuclei images at 40× due to the close proximity and intricate details, hindering accurate differentiation and learning. These limitation is extended as future work.

### Future work

- The lack of a comprehensive dataset of histopathology images presents significant challenges for the integration of real-time clinical datasets in the future.
- To address the challenges^32,33^, a new model will be proposed to tackle the issue of overlapping nuclei in whole slide images a new technique introduced like CCP to enhance image quality and address issues related to staining, noise, and various artifacts, including scratches and air bubbles.
- The new proposed model will employ to employ Cell Classification and Processing method in conjunction with a nuclei detection model, such as HoverNet fusion CNN, for classifying mitosis. This approach aims to facilitate faster cancer grading while reducing computational time.
- Additionally, the segmentation of images with high nuclei density will be addressed, along with the introduction of new techniques to enhance processing efficiency and intelligence through improved input methods.
- Furthermore, the methodology will be expanded to include whole slide images in histopathology for various types of cancer, including lung, colon, and skin cancers.
- Future studies will focus on classification models, particularly ensemble model fusion with attention mechanisms, to design efficient learning processes. Advanced techniques and testing methods will also be incorporated to improve model outcomes^27,28^.

## Data Availability

https://web.inf.ufpr.br/vri/databases/breast-cancer-histopathological-database-breakhis/. https://github.com/suriyabegum/suriya.
